# Gas1-high quiescent neural stem cells are multipotent and produce oligodendrocytes during aging and after demyelinating injury

**DOI:** 10.1371/journal.pbio.3003100

**Published:** 2025-04-03

**Authors:** Chaoqiong Ding, Zhenzhong Pan, Xiang Yan, Ran Zhou, Huifang Li, Lu Chen, Yuan Wang, Yan Zhang

**Affiliations:** 1 National Clinical Research Center for Geriatrics, State Key Laboratory of Biotherapy, West China Hospital, Chengdu, China; 2 Tianfu Jincheng Laboratory, Chengdu, China; 3 Department of Neurosurgery, State Key Laboratory of Biotherapy and Cancer Center, West China Hospital, Sichuan University, Chengdu, China,; 4 Core Facilities of West China Hospital, Sichuan University, Chengdu, China; 5 Key Laboratory of Birth Defects and Related Diseases of Women and Children of MOE, State Key Laboratory of Biotherapy, West China Second Hospital, Sichuan University, Chengdu, China; TU Munich: Technische Universitat Munchen, GERMANY

## Abstract

Quiescent neural stem cells (qNSCs) in the adult mouse subventricular zone (SVZ) normally have limited capacity to generate glia. Gliogenic domains are present in both dorsal and ventral SVZ, with the ventral region featuring a subpopulation of Gli1^+^ qNSCs. In dorsal SVZ, however, the molecular identity and developmental origin of oligodendrogenic qNSCs remains elusive. Here, through single-cell analysis and lineage tracing, we identify an undefined subpopulation of Gas1^high^ qNSCs in dorsal SVZ, distinct from Gli1^+^ qNSCs. These cells originate from embryonic Gas1^high^ dorsal radial glia, and persist into the aged SVZ. Remarkably, they are multipotent and more gliogenic than Gas1^low/−^ qNSCs, continuously generating oligodendrocytes in the adult and aged brain, and can be mobilized for myelin repair upon demyelination. Together, our study uncovers a subpopulation of dorsally derived, multipotent long-term qNSCs in the adult and aged SVZ with enhanced gliogenic potential, shedding light on the heterogeneity and plasticity of NSCs in normal, aging, and disease conditions.

## Abstract 2

Quiescent neural stem cells (qNSCs) in the adult mouse subventricular zone are known to contain a limited capacity to generate glia. In this study, the authors identify a subpopulation of multipotent qNSCs, distinguished by high Gas1 expression, with a higher propensity to produce oligodendrocytes throughout life and after demyelinating injury.

## Introduction

Adult neural stem cells (NSCs) in the subventricular zone (SVZ) (also named B cells) of the rodent brain continuously generate new neurons and glial cells throughout life [1]. These cells originate from a distinct subpopulation of embryonic radial glia (RG) entering a slow-cycling state between E13.5 and E15.5, which also generate ependymal cells (EPCs) lining up the ventricular wall [2–5]. Previous studies have identified several regulators essential for the embryonic-to-adult NSC transition using candidate approaches, including cell cycle/DNA replication inhibitor p57, Geminin, and cell adhesion molecule Vcam1 [2,4,5]. However, whether these regulators are transiently or persistently required has not been defined, and it is unknown whether other players are involved in driving this important transition.

Instead of being a homogeneous population, adult SVZ NSCs are comprised of multiple subpopulations with unique gene expression profiles, positional identity, and lineage differentiation potential. Based on their activation states, NSCs can be divided into quiescent NSCs (qNSCs) and activated NSCs (aNSCs) [6], whose molecular heterogeneity has been well characterized by single-cell RNA sequencing (scRNA-seq) analysis [7–9]. Cultured NSCs can be differentiated into all three lineages (neuron, astrocyte, and oligodendrocyte) in vitro [1]. However, under physiological conditions in vivo, the majority of adult SVZ NSCs generate transit-amplifying progenitors (TAPs) and neuroblasts (NBs), which migrate along the rostral migratory stream into the olfactory bulb (OB) to generate diverse subtypes of neurons [1], depending on the regional identities of NSCs in the SVZ [10,11].

In contrast, only a minority of adult NSCs in the SVZ can give rise to oligodendrocytes in the corpus callosum (CC) [12,13], which is regulated by Sonic hedgehog signaling [12,14,15]. In the ventral SVZ, it has been shown that there is a subpopulation of Shh-responsive, Gli1^+^ qNSCs that persist into the aged brain and can generate oligodendrocytes in vivo [16]. Additionally, these Gli1^+^ NSCs can also be mobilized in response to demyelination, to generate a large number of oligodendrocytes for myelin repair [17]. In the dorsal SVZ, however, the molecular identity of adult NSCs that are capable of generating oligodendrocytes in normal, aging, and diseased conditions is largely unknown. The level of Shh signaling is transiently high in the dorsal SVZ at early postnatal stages leading to mass production of oligodendrocytes from dorsal NSCs, but is dramatically reduced in the dorsal areas and restricted to the ventral SVZ in adult brains [18–20]. Recently, it was reported that deletion of PDGFRβ releases qNSCs from quiescence to become gliogenic in multiple domains, including the dorsolateral and dorsoseptal regions of the SVZ [21]. However, PDGFRβ is expressed in the majority of qNSCs, as well as ~50% of aNSCs [21]. Thus, whether a specific subpopulation of qNSCs is oligodendrogenic in the dorsal SVZ of the adult and aged brain remains to be determined.

In this study, combining single-cell analysis, lineage tracing, and functional validation, we identify a previously undefined subpopulation of Gas1^high^ adult dorsal NSCs with minimal *Gli1* expression, which originates from embryonic RG and persist into the aged brain, continuously generating oligodendrocytes and actively contributing to myelin repair.

## Results

### Gas1 is dynamically expressed during the transition from embryonic RG to adult SVZ NSCs

**Fig 1:**
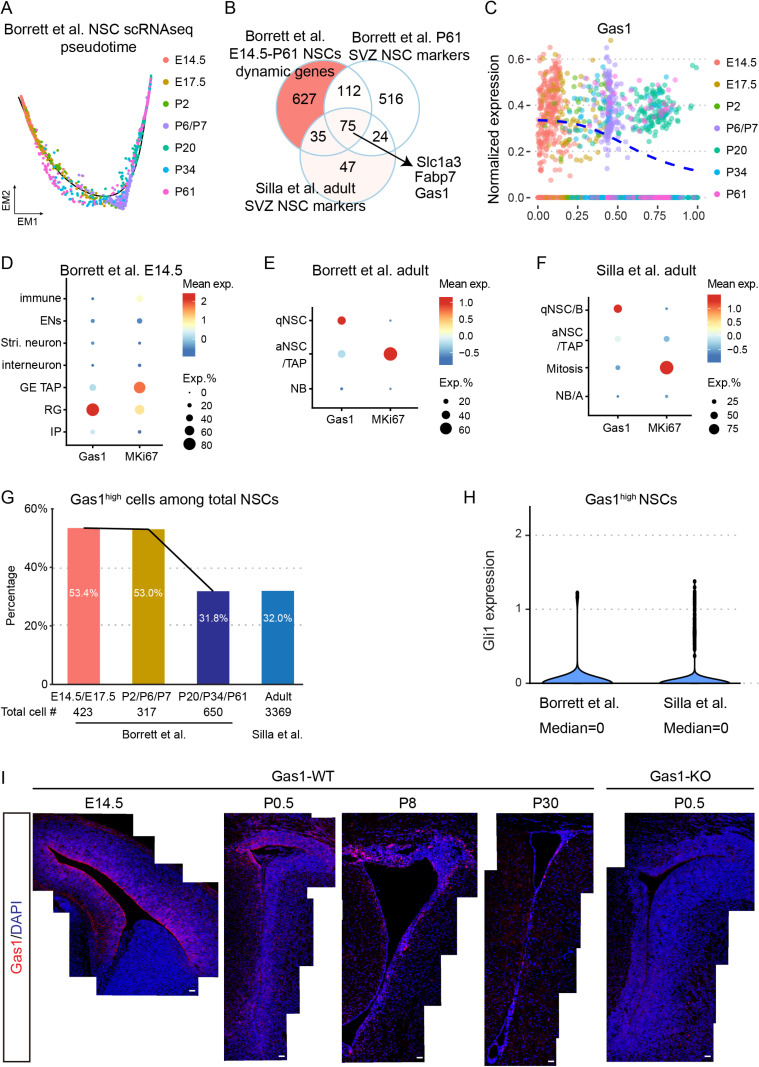
Dynamic expression of Gas1 in NSCs during development and adulthood. **(A)** Pseudotime ordering of NSCs at different timepoints (color-coded) from Borrett and colleagues dataset. Total cell numbers: E14.5 RG (*n* =  333 cells), E17.5 RG (*n* =  90 cells), P2 NSCs (*n* =  47 cells), P6/P7 qNSCs (*n* =  270 cells), P20 NSCs (n =  415 cells), P34 NSCs (*n* =  106 cells), and P61 NSCs (*n* =  129 cells). **(B)** Venn diagram showing the intersection of dynamic genes identified in pseudotime analysis, P61 SVZ NSCs signature genes, and Silla and colleagues adult SVZ NSCs signature genes. **(C)** Scatter plots showing the expression of Gas1 in NSCs along the pseudotime trajectory. **(D–**F) Dot plots displaying Gas1 and Mki67 expression in Borrett and colleagues E14.5 VZ/SVZ cells, as well as adult SVZ cells (Borrett and colleagues P20/P34/P61 and Silla and colleagues datasets). (G) Bar charts comparing the percentages of Gas1high NSCs (Gas1 expression >1) across different timepoints. **(H)** Violin chart of Gli1 expression in Gas1high NSCs from Borrett and colleagues P20/P34/P61 and Silla and colleagues datasets. **(I)** Panoramic images of brain coronal sections stained with Gas1 antibody at different developmental time points in mice. P0 Gas1 KO mice served as the negative control for Gas1 IF staining. Scale bars, 100 μm.

To systematically explore the transcriptional regulation of the transition from embryonic RG to adult NSCs, we reanalyzed the mouse VZ/SVZ scRNAseq data from Borrett and colleagues study [8], encompassing developmental stages from embryonic (E14.5/E17.5) to postnatal (P2/P6/P7) and adult (P20/P34/P61) (S1 and S2A Figs). We isolated E14.5-P61 NSCs in silico, performed pseudotime analysis [22] (Fig 1A), and identified genes that are dynamically expressed along the embryonic-to-adult NSC trajectory (hereafter, dynamic genes) (S1 Table). Notably, the expression of the classic RG marker *Blbp* (*Fabp7*) and proliferation marker *Mki67* is dramatically reduced along the trajectory (S2C Fig), consistent with the shift from proliferative embryonic NSCs to quiescent adult stem cells. *Geminin* (*Gmnn*), *p57* (*Cdkn1c*), and *Vcam1*, previously implicated in this developmental transition [2–4], are also dynamically expressed. While *Vcam1* maintains expression in adult NSCs, both *Geminin* and *p57* exhibit minimal expression, suggesting that their roles are transient during embryonic development (S2D Fig).

To identify genes with enduring influence on the embryonic-to-adult NSC transition, we overlap dynamic genes with genes uniquely expressed in NSCs relative to more differentiated cell types within the adult SVZ (Fig 1B). We further validated the results using the Silla and colleagues adult SVZ scRNA-seq dataset [9] (S2B Fig), and identified 75 candidate genes (Fig 1B and S2 Table). Among the top genes, we found *Growth Arrest-Specific-1* (*Gas1*) of particular interest. Initially named after its role in growth suppression [23], Gas1 was later recognized as a co-receptor for Patched1 that promotes SHH signaling [24,25], whose role in RG and adult NSCs have not been reported. The expression of *Gas1* is high in embryonic RG but decreases in adult NSCs with low *Mki67* expression, yet it remains an NSC-specific marker at both stages (Fig 1C–1F). The decline of the average *Gas1* expression in adult NSCs can be attributed to a lower proportion of NSCs expressing *Gas1* at high levels (normalized expression >1, hereafter, Gas1^high^), dropping from 53.4% during embryonic stages to 31.8%−32% in adulthood (Fig 1G). Intriguingly, despite Gas1’s role as an activator of SHH signaling, Gas1^high^ NSCs in the adult SVZ exhibit minimal *Gli1* expression (Gli1^min^) (Fig 1H), suggesting they are distinct from the previously described ventral Gli1^+^ NSCs [16]. Together, developmental trajectory analysis highlights *Gas1* as a candidate potentially implicated in the transition from embryonic to adult NSCs, whose high-level expression is enriched in a subset of adult Gli1^min^ NSCs.

To confirm the temporal dynamics and regional specificity of Gas1 expression, we performed immunofluorescence on coronal brain sections at different timepoints (E14.5, P0.5, P8, and P30) with Gas1 antibody staining (Fig 1I). At E14.5, Gas1 mainly labels RG in the dorsal and septal VZ/SVZ (Fig 1I). In postnatal and young adult brains, it is a marked reduction of Gas1 expression in the VZ and SVZ regions (Fig 1I). At P30, Gas1 expression is mainly found in the dorsolateral SVZ, with minimal expression in the surrounding brain areas including the CC (Fig 1I). In contrast, there is minimal Gas1 staining in the Gas1-null brain, which validates the specificity of the Gas1 antibody (Fig 1I). These data confirm the temporal dynamics of Gas1 revealed by pseudotime analysis, supporting the notion that Gas1 expression is mainly restricted in the VZ and SVZ.

### Dorsal Gas1^high^ RG at E14.5 give rise to postnatal SVZ NSCs

**Fig 2:**
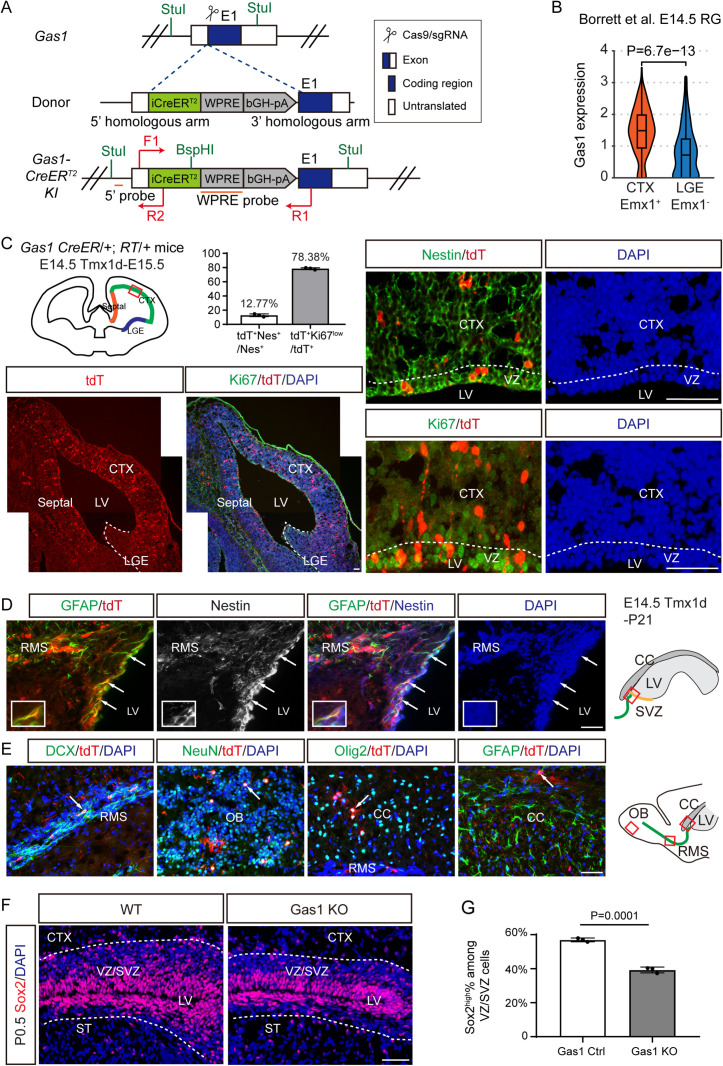
Lineage tracing of Gas1^high^ RGs at E14.5. **(A)** Schematic for the knock-in strategy for *Gas1* CreER/ + mice. E1, exon 1 of *Gas1*. StuI and BspHI, restriction sites. 5′ probe and WPRE probe, probes for Southern blot. F1, R1, and R2, PCR primers for genotyping. **(B)** Violin chart of *Gas1* expression in Borett and colleagues E14.5 CTX (Emx1 high-level cluster) RG and LGE (Emx1 low-level cluster) RG, compared by Wilcoxon test. **(C)** Top left: Schematic showing the Tmx induction and analysis strategy. *Gas1* CreER/+; RT/+ mice were Tmx-induced at E14.5 (50 μg/g body weight) and analyzed at E15.5 using coronal brain sections. Top right: Representative IF co-labeling of Nestin and tdT (DsRed) in the E15.5 VZ/SVZ. Bottom panels: Representative IF co-labeling of Ki67 and tdT (DsRed) at low (left) and high (right) magnifications. The dashed line roughly marks the LGE (bottom left) or the VZ (bottom right). **(D)** *Gas1* CreER/+; RT/+ mice were Tmx-induced at E14.5 and analyzed at P21 using sagittal brain sections. Representative IF co-labeling of GFAP, tdT (DsRed), and Nestin in the P21 SVZ are shown. Arrows highlight examples of GFAP^+^tdT^+^Nestin^+^. **(E)** Representative IF co-labeling of DCX, NeuN, or GFAP with tdT in the RMS, OB, or CC at P21, respectively. Arrows highlight examples of colocalizing cells. **(F)** Representative Sox2 IF staining in the VZ/SVZ of wild-type and *Gas1* KO mice at P0.5. The dashed lines roughly mark the boundary of VZ/SVZ. **(G)** Quantification of the percentage of Sox2^high^ cells among all DAPI^+^ cells in the VZ/SVZ in (F). *n* =  3 mice for each group. Data represent Mean ±  SD. CTX, cortex; LGE, lateral ganglionic eminence; LVs, lateral ventricles; VZ, ventricular zone; SVZ, subventricular zone; ST, striatum; RMS, rostral migratory stream; CC, corpus callosum; OB, olfactory bulb. Scale bars, 100 μm. The original data underlying Fig 2C and 2G can be found in S1 Data.

To fate-map Gas1^high^ NSCs across developmental stages, we used CRISPR-Cas9-mediated genome-editing to knock-in (KI) the CreER^T^2 sequence into the exon 1 locus of mouse *Gas1* gene, and established a tamoxifen (Tmx)-inducible Cre line under the endogenous *Gas1* promoter (hereafter, *Gas1* CreER/+) (Fig 2A). Notably, this KI strategy results in loss of one wild-type *Gas1* allele. We confirmed the genotypes by PCR and performed Southern blot to validate targeted insertion (S3A and S3B Fig). By crossing to Ai9 Rosa-CAG-LSL-tdTomato (RT) reporter mice [26], we established *Gas1* CreER/+; RT/+ mice and examined the fidelity of the Cre in the adult SVZ. Indeed, the majority of Gas1-expressing cells are readily labeled by tdT after 5× Tmx-treatment from P26 to P30 (S3C Fig).

To determine the fate of Gas1^high^ RG at E14.5, we treated pregnant females at E14.5 with a single low dose of Tmx (50 μg/g body weight). Based on scRNA-seq analysis, at E14.5 *Gas1* is enriched in the dorsal cortical Emx1^+^ RG compared to Emx1^−^ RG from the lateral ganglionic eminence (LGE) [8,27] (Fig 2B). Consistently, when analyzed at E15.5, tdTomato-positive (tdT^+^) cells are restricted in radial columns in the dorsal cortical and septal regions, but not the LGE (Fig 2C). The dorsal/septal distribution of tdT^+^ cells is consistent with the pattern of Gas1 antibody staining (Fig 1I). These tdT^+^ cells include Nestin^+^ RG that remain in the VZ, as well as Ki67^−^ cells with migratory neural progenitor morphology in the cortex likely differentiated from E14.5 Gas1^high^ RG (Fig 2C). At P21, E14.5-labeled Gas1^high^ RG has transformed into GFAP^+^Nestin^+^ adult NSCs in the dorsal anterior SVZ (Fig 2D). Postnatal SVZ NSCs and EPCs have shared embryonic origin from E13.5 to E15.5 RG [4,5]. Consistently, we could identify tdT^+^ cells that express the EPC marker Foxj1 along the lateral ventricle (LV) wall (S3D Fig). Overall, tdT marks 43% of total NSCs (212/492 NSCs) and 6.9% of total EPCs (20/289 EPCs, adjacent sections) at P21 (S3D Fig). Besides, tdT also marks differentiated progeny of postnatal SVZ NSCs including DCX^+^ NBs in the RMS and NeuN^+^ neurons in the OB, as well as Olig2^+^ oligodendrocyte lineage cells and less frequently GFAP^+^ astrocytes in the CC (Fig 2E).

Trajectory analysis indicates that *Gas1* is also highly expressed in a subset of postnatal NSCs in the SVZ at P6/P7 (Fig 1C). To determine whether these Gas1^high^ cells can serve as precursors of adult NSCs, we treated the *Gas1* CreER/+; RT/+ mice with a single dose of Tmx at P8 and analyzed at P30. In the anterior dorsal SVZ, ~ 67% tdT^+^ cells are GFAP^+^/Nestin^+^ adult NSCs (143/214, *n* = 3 mice) at P30 (S4A Fig), while the remaining tdT^+^ cells are mostly neural progenitor cells. A large number of tdT^+^ NBs and neurons are found in the rostral RMS and OB, respectively (S4B Fig). We also observed clusters of Olig2^+^ oligodendrocyte lineage cells and GFAP^+^ astrocytes in the CC (S4C and S4D Fig), consistent with the gliogenic potential of dorsal SVZ NSCs at P8 [18].

To determine whether Gas1 is essential for the transition from embryonic RG to postnatal NSCs, we generated *Gas1* homozygous knock-out mice (*Gas1* KO) with the genotype *Gas1* CreER/CreER. Consistent with previous reports [24,28], *Gas1* KO mice exhibited small eyes and died shortly after birth. We compared the number and proportion of NSCs expressing high-level Sox2 in the VZ/SVZ from wildtype and *Gas1* KO mice at P0.5. Both the number and proportion of Sox2^high^ NSCs are reduced in the *Gas1* KO VZ/SVZ (Fig 2F and 2G), supporting the notion that Gas1 regulates the generation of postnatal NSCs [29].

### Gas1^high^ adult SVZ NSCs are multipotent and exhibit enhanced oligodendrogenic potential

**Fig 3:**
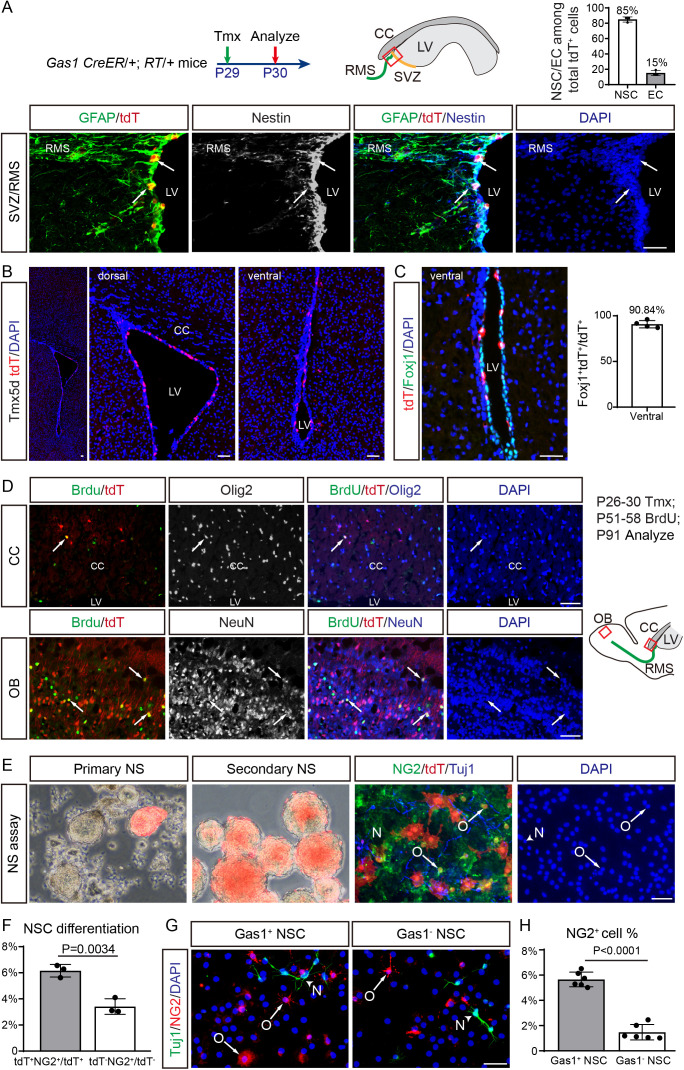
Gas1^high^ adult SVZ NSCs are multipotent and more oligodendrogenic than Gas1^low^/Gas^−^ NSCs. **(A)** *Gas1* CreER/ + ; RT/ + mice were Tmx-induced at P29 (125 μg/g body weight) and analyzed at P30 using sagittal brain sections. Representative IF co-labeling of GFAP, tdT (DsRed) and Nestin in the P30 SVZ are shown. Arrows highlight examples of GFAP^+^tdT^+ ^Nestin^+ ^. Quantification of the percentage of NSCs or ECs among total tdT^+^ cells in the SVZ. *n* =  3 mice. Data represent Mean ±  SD. **(B)** *Gas1* CreER/ + ; RT/ + mice were Tmx-induced from P26 to P30 and analyzed at P31 using coronal brain sections. Representative images show the overall the distribution of Gas1^high^ tdT^+^ cells. **(C)** Representative coronal IF co-labeling of Foxj1 and tdT in the ventral of LV. Quantification of the percentage of Foxj1^+^ tdT^+^ cells among total tdT^+^ cells in the ventral of LV. *n* =  4 mice. Data represent Mean ±  SD. **(D)** *Gas1* CreER/ + ; RT/ + mice were 5× Tmx-induced from P26 to P30, fed with BrdU in drinking water (0.8 mg/ml) from P51 to P58, and analyzed at P91 using sagittal brain sections. Representative IF co-labeling of BrdU/tdT with Olig2 or NeuN in the P91 CC or OB are shown. Arrows highlight examples of colocalizing cells. **(E)** Left to right: The SVZ from P26 to P30 Tmx-induced *Gas1* CreER/ + ; RT/ + mice were dissected and cultured as primary neurospheres (NS). Red primary NS with tdT were picked up by pipette before passage to enrich tdT^+^ NSCs in secondary NS culture. Mixed tdT^+^ and tdT^−^ NSCs were differentiated in the same wells, and IF stained for NG2, Tuj1 and tdT (DsRed). Arrows highlight examples of tdT^+ ^NG2^+^ OPCs (O), while arrowheads indicate tdT^+ ^Tuj1^+^ immature neurons (N). **(F)** Quantification of the percentage of tdT^+ ^NG2^+^ OPCs among total tdT^+ ^DAPI^+^ cells, and tdT^−^NG2^+^ OPCs among total tdT^−^DAPI^+^ cells in the NSC differentiation assay in (E). *n* =  3 biological replicates. Data represent Mean ±  SD. **(G)** Cultured NSCs were separated into Gas1^+^ and Gas1^−^ subpopulations by antibody-conjugated magnetic beads, differentiated in different wells, and IF stained for NG2 and Tuj1. Arrows highlight NG2^+^ OPCs (O), while arrowheads indicate Tuj1^+^ immature neurons (N). **(H)** Quantification of the percentage of NG2^+^ OPCs among total DAPI^+^ cells differentiated from Gas1^+^ or Gas1^−^ NSCs in (G). *n* =  6 biological replicates for each group. Data represent Mean ±  SD. LVs, lateral ventricles; SVZ, subventricular zone; RMS, rostral migratory stream; CC, corpus callosum; OB, olfactory bulb. Scale bars, 100 μm. The original data underlying Fig 3A, 3C, 3F, and 3H can be found in S1 Data.

We next investigated the properties of Gas1^high^ cells in the adult SVZ of *Gas1* CreER/+; RT/+ mice. A single Tmx pulse at P29 specifically labels Gas1^high^ NSCs and a small population of EPCs at P30 (~85% GFAP^+^Nestin^+^ and ~15% Foxj1^+^ among total tdT-labeled cells) (Fig 3A), but not in more differentiated cells such as NBs, OPCs/oligodendrocytes or astrocytes. Notably, 5× Tmx-treatment from P26 to P30 increases the number of labeled tdT^+^GFAP^+^Nestin^+^ NSCs (1× Tmx: 16.7 cells/section; 5× Tmx: 23.8 cells/section) (S3C Fig). We imaged the entire SVZ to show the overall the distribution of Gas1^high^ tdT^+^ cells, the majority of which are in the dorsal SVZ (Fig 3B). Similar to antibody staining, we did not observe tdT^+^ cells in the CC (Figs 3B and 1I). For the few tdT^+^ cells in the ventral SVZ, we double-checked their identity and found that they are mostly (~90%) Foxj1^+^ EPCs (Fig 3C).

Under physiological conditions, few adult SVZ NSCs give rise to oligodendrocyte lineage cells in the CC [12]. Remarkably, 2 months after P26–P30 Tmx-treatment, we frequently observed small clusters of Olig2^+ ^tdT^+^ cells in the CC (2.6 cells/section), in addition to differentiated NeuN^+ ^tdT^+^ neurons in the OB (Fig 3D). A subset of the tdT^+^ cells in the CC and OB can be labeled by BrdU pulse-chase assay from P51–P58 to P91, indicating that they are newly differentiated oligodendrocytes or neurons (Fig 3D). These data demonstrate that Gas1^high^ NSCs continuously contribute to both oligodendrogenesis and neurogenesis in the adult brain.

To directly compare the oligodendrogenic potential of Gas1^high^ versus Gas1^low^/Gas1^−^NSCs, we cultured P31 SVZ cells after P26–P30 Tmx-induction. Consistent with their NSC identity, tdT^+^ cells could readily form primary and secondary neurospheres under free-floating NSC culture conditions (Fig 3E). Under the NSC differentiation condition, tdT^+^ NSCs generate twice as many NG2^+^ OPCs as tdT^−^ NSCs cultured in the same well (Fig 3E and 3F). We further confirmed these results by isolating Gas1^+^ NSCs from Gas1^−^ NSCs using antibody-conjugated magnetic beads, allowing them to independently undergo differentiation. The purity of magnetically sorted Gas1^+^ NSCs are confirmed by FACS analysis (S5A and S5B Fig). Consistently, we found increased ratio of NG2^+^ OPCs differentiated from Gas1^+^ NSCs compared to those from Gas1^−^ NSCs (Fig 3G and 3H). These results collectively indicate that Gas1^high^ adult SVZ NSCs are multipotent in vivo and exhibit enhanced oligodendrogenic potential compared to Gas1^low^/Gas1^−^ NSCs.

**Fig 4:**
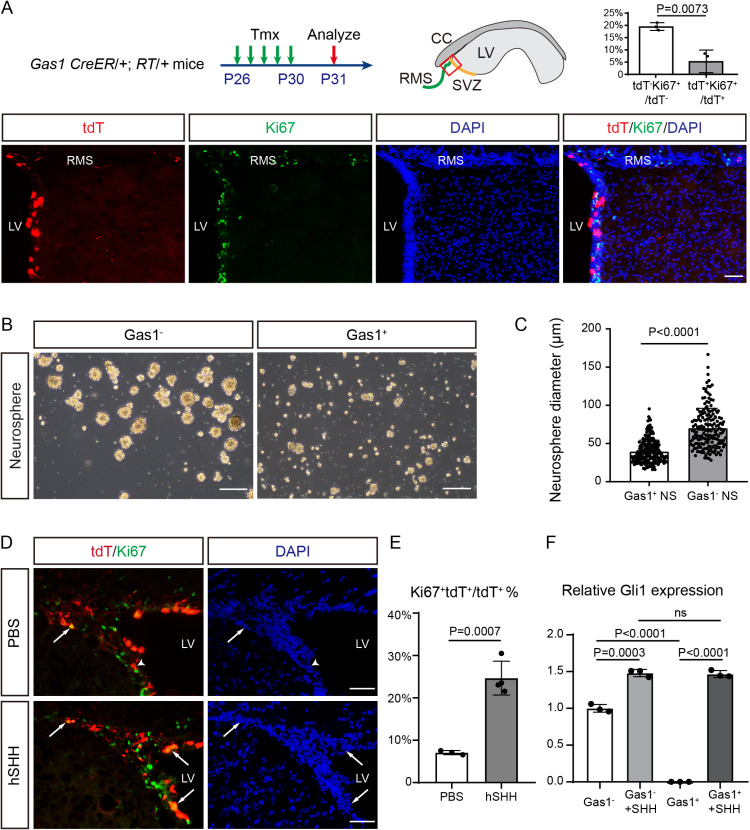
Gas1^high^ adult NSCs are relatively quiescent but can be activated by SHH. **(A)** *Gas1* CreER/ + ; RT/ + mice were Tmx -induced from P26 to P30 and analyzed at P31 using sagittal brain sections. Representative sagittal IF co-labeling of Ki67 and tdT in the dorsal anterior SVZ at P31 are shown. Scale bars, 100 μm. Quantification of the percentage of Ki67^+^ tdT^−^ cells among total tdT^−^ cells and Ki67^+^ tdT^+^ cells among total tdT^+^ cells. *n* =  3 mice. Data represent Mean ±  SD. **(B)** Representative images of MACS-Gas1^−^ cells and MACS-Gas1^+^ cells cultured into neurospheres in vitro at 7 days. Scale bars, 250 μm. **(C)** Quantification of the average diameter of MACS-Gas1^−^ neurospheres (*n* =  174 NS) and MACS-Gas1^+^ neurospheres (*n* =  224 NS). Data represent Mean ±  SD. **(D)** Representative IF co-labeling of Ki67 and tdT in the SVZ. Arrows highlight examples of Ki67^+ ^tdT^+^ NSCs. The Ki67^−^tdT^+^ NSCs are indicated by an arrowhead. Scale bars, 100 μm. **(E)** Quantification of the percentage of Ki67^+ ^tdT^+^ cells among total tdT^+^ cells in the SVZ from PBS-injected (*n* =  3 mice) and hSHH-injected group (*n* =  4 mice). Data represent Mean ±  SD. **(F)** The relative expression level of Gli1 in different groups. *n* =  3 biological replicates, Data represent Mean ±  SD. The original data underlying Fig 4A, 4C, 4E, and 4F can be found in S1 Data.

We further investigate the potential roles of Gas1 in NSCs. Overexpressing *Gas1* in cultured NSCs inhibits cell cycle transition from G0/G1 phase to S phase in the EdU labeling assay (S5C and S5D Fig), while Gas1 KO NSCs exhibited increased S phase ratio (S5E and S5F Fig), consistent with the growth suppressive function of Gas1 [23]. Pseudotime analysis of adult SVZ differentiation trajectory also indicates that Gas1 is negatively correlated with Mki67 (S5G Fig). Indeed, the Ki67% of tdT^+^ cells are much lower than tdT^−^ cells (Fig 4A), and the sphere size of Gas1^+^ NSCs are smaller than Gas1^−^ NSCs (Fig 4B and 4C). On the other hand, Gas1^high^ NSCs labeled by tdT in the adult SVZ dramatically increase their proliferation upon intraventricular human SHH injection (Fig 4D and 4E), and exhibits increased Gli1 expression after SHH treatment (Fig 4F). These data indicate that while Gas1^high^ NSCs express minimal *Gli1* under physiological conditions, they remain responsive to external SHH. This may explain its enhanced oligodendrogenic capacity given the key role of SHH in oligodendrogenesis [14,18].

### Gas1^high^ adult NSCs persist into the aged brain

**Fig 5:**
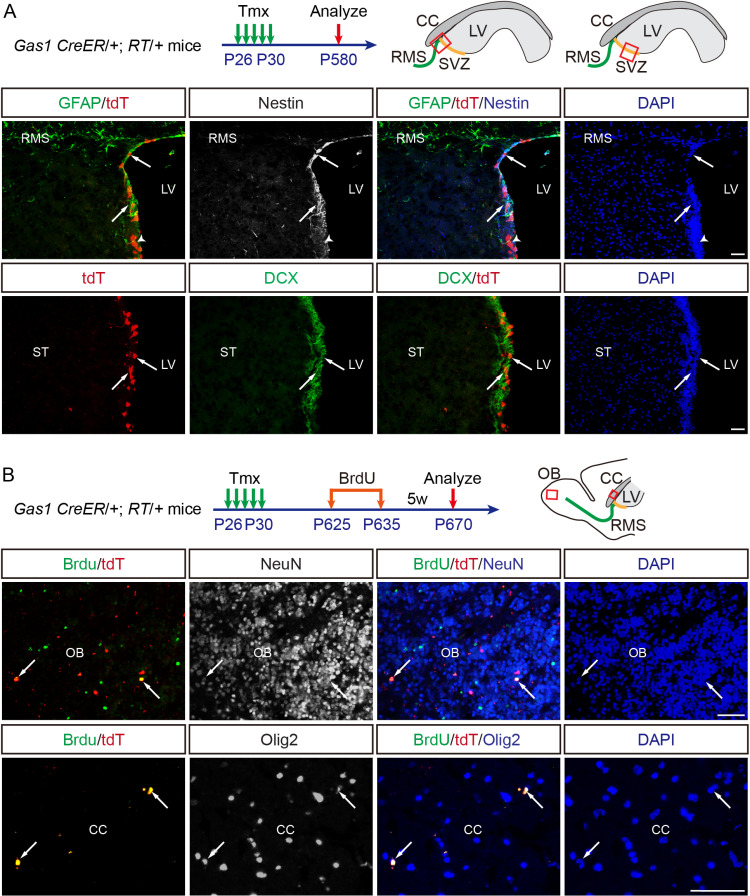
Gas1^high^ adult NSCs persist into the aged brain and generate newborn neurons and oligodendrocyte lineage cells. **(A)** *Gas1* CreER/ + ; RT/ + mice were Tmx-induced from P26 to P30 and analyzed at P580 using sagittal brain sections. Representative IF co-labeling of GFAP, tdT and Nestin or IF co-labeling of DCX and tdT in the P580 SVZ are shown. **(B)** *Gas1* CreER/+; RT/+ mice were Tmx-induced from P26 to P30, fed with BrdU in drinking water (0.8 mg/ml) from P625 to P635, and analyzed at P670 using sagittal brain sections. Representative IF co-labeling of BrdU/tdT with NeuN or BrdU/tdT with Olig2 in the P670 OB or CC are shown. Arrows highlight colocalizing cells, and arrowheads indicate tdT^+^GFAP^−^Nestin^−^ cells. LVs, lateral ventricles; SVZ, subventricular zone; RMS, rostral migratory stream; CC, corpus callosum; OB, olfactory bulb; ST, striatum. Scale bars, 100 μm.

To determine whether Gas1^high^ NSCs represent a subpopulation of long-term NSCs, we labeled these cells from P26 to P30, and analyzed their fate at 19 months of age (P580). In these aged brains, we consistently identified GFAP^+ ^/Nestin^+ ^/tdT^+^ NSCs in the SVZ, as well as their derivative Dcx^+ ^/tdT^+^ NB in the SVZ and RMS (Fig 5A). BrdU pulse-chase assay from P625–P635 to P670 further confirms that GFAP^+ ^/Nestin^+ ^/tdT^+^ NSCs and their derivative progenitor cells continue to generate NeuN^+ ^/BrdU^+ ^/tdT^+^ newborn neurons in the OB and Olig2^+ ^/BrdU^+ ^/tdT^+^ in the CC at this stage (Fig 5B).

**Fig 6:**
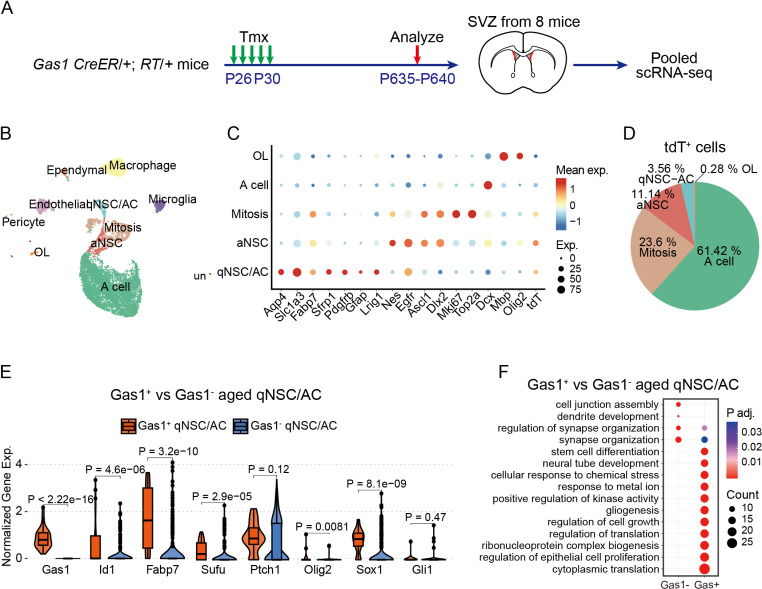
Single-cell analysis reveals the upregulation of gliogenesis pathway genes in Gas1high NSCs from the aged SVZ. **(A)** *Gas1* CreER/+; RT/+ mice were Tmx-induced from P26 to P30 and collected at P635 for single-cell sequencing and analysis. **(B)** UMAP plot of high-quality cells from SVZ of *Gas1* CreER/+; RT/+ aged mice (*n* =  11,081 cells pooled from 8 mice). Colors distinguish different cell types, defined by marker genes used in Silla and colleagues and Borrett and colleagues studies. **(C)** Dot plot of the expression level and percentage of marker genes along with Gas1 and tdT in different cell types. **(D)** Pie chart showing the cell-type composition among tdTomato-labeled cells (*n* =  1,069 cells). **(E)** Violin charts of Id1, Fabp7, Sufu, Ptch1, Olig2, Sox1, and Gli1 expression in Gas1^+^ qNSCs (Gas1 expression > 0, *n* =  26 cells) and Gas1-qNSCs (Gas1 expression =  0, *n* =  230 cells), compared by Wilcoxon test. **(F)** Bubble plots of significantly upregulated (UP) or downregulated (Down) pathways in Gas1^+^ qNSCs vs. Gas1^−^ qNSCs (*P* adj. <  0.05), based on Gene Ontology enrichment of differentially expressed genes (|avg_log2FC | >  0.5, *P* <  0.05).

To dissect the cellular identities and molecular profiles of Gas1^high^ NSCs in aged SVZ, we pooled the dorsal lateral SVZ tissue from eight aged mice at 21 months of age (P635–P640), and performed scRNA-seq analysis (Fig 6A). We obtained 11,081 high quality cells, which were clustered into 11 cell types characterized by cell-type-specific markers (Fig 6B and 6C and S3 Table). Among all the tdT^+^ cells in the SVZ, the majority of them reside along the neurogenesis axis of qNSC-aNSC/TAP-Mitosis-NB, while a small proportion of them are oligodendrocytes (Fig 6D). Compared to Gas1^−^ cells, Gas1^+^ cells express higher level of qNSC markers (*Id1* and *Fabp7*), Shh pathway gene (*Sufu*) and gliogenesis/neurogenesis transcription factors (*Olig2* and *Sox1*) (Fig 6E). Notably, there is minimal *Gli1* expression in either Gas1^+^ or Gas1^−^ qNSCs (Fig 6E). Gene ontology analysis further reveals that Gas1^+^ cells up-regulate translation, neurogenesis and gliogenesis pathways (Fig 6F). Together, these data demonstrate that Gas1^high^ NSCs in the young adult SVZ persist into the aged brain, and continue to generate differentiated neurons and glia.

### Gas1^high^ adult NSCs actively contribute to myelin repair after cuprizone-induced demyelination

**Fig 7:**
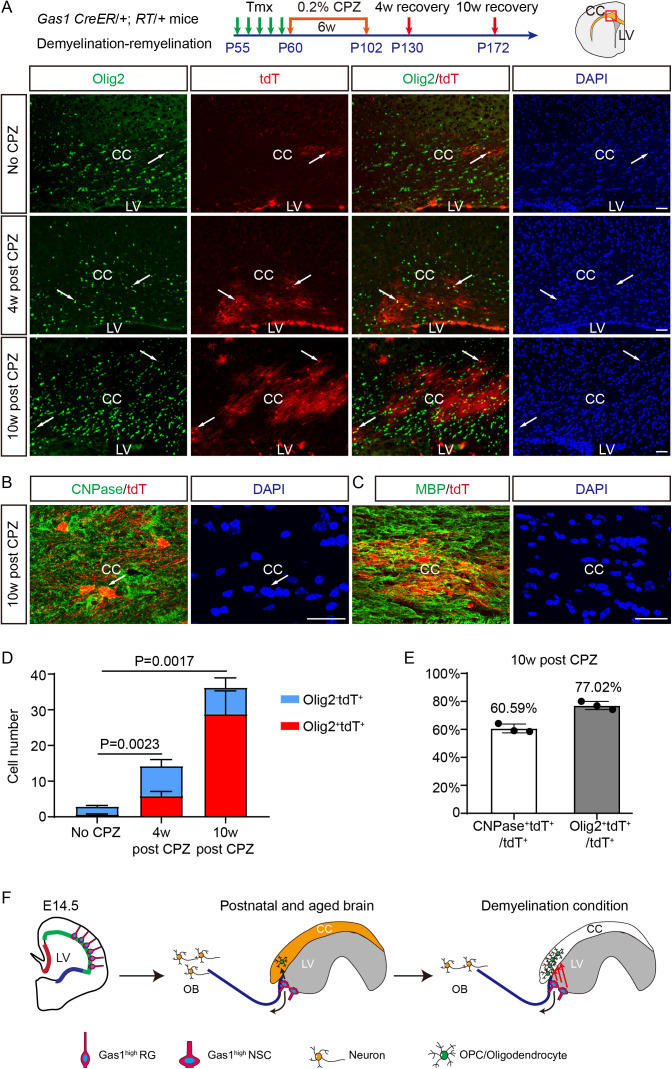
Gas1^high^ adult NSCs contribute to myelin repair after cuprizone-induced demyelination. **(A)** *Gas1* CreER/+; RT/+ mice were tamoxifen-induced from P55 to P59, fed with 0.2% CPZ in diet from P60 to P102, and analyzed at 4 or 10 weeks later. Coronal brain sections from the control diet group (No CPZ), 4-week recovery group (4 weeks post-CPZ), and 10-week recovery group (10 weeks post-CPZ) were IF stained for Olig2 and tdT. Arrows highlight examples of colocalizing cells. **(B)** Representative confocal IF co-labeling of CNPase and tdT in the CC from the 10-week recovery group. Arrows highlight examples of colocalizing cells. **(C)** Representative confocal IF co-labeling of MBP and tdT in the CC from the 10-week recovery group. Arrows highlight examples of colocalizing cells. **(D)** The number of total tdT^+^ cells per section and proportion of Olig2^+ ^tdT^+^ and Olig2^−^tdT^+ ^cells among total tdT^+^ cells in the CC. The *P*-values represent the comparison of Olig2^+ ^tdT^+^ cell number between each group. *n* = 3 mice for each group, 6–12 sections for each mouse. Data represent Mean ±  SD. **(E)** The percentage of CNPase^+^tdT^+^ and Olig2^+ ^tdT^+^ cells among total tdT^+^ cells in the CC from the 10-week recovery group. *n* = 3 mice, 3–5 sections for each mouse. Data represent Mean ±  SD. LVs, lateral ventricles; CC, corpus callosum; CPZ, cuprizone. Scale bars, 100 μm. **(F)** Schematic summarizing the developmental origin and function of the Gas1^high^ NSC subpopulation. The original data underlying Fig 7D and 7E can be found in S1 Data.

Given Gas1^high^ NSCs are long-term NSCs with enhanced oligodendrogenic potential, we investigated whether they could be mobilized for myelin repair in response to demyelination. We induced demyelination in the CC through 6-week treatment with dietary cuprizone (CPZ) [17,30]. Using Luxol Fast Blue staining, we confirmed the demyelination immediately after CPZ treatment and the gradual remyelination at 4 and 10 weeks after CPZ removal (S6A and S6B Fig). Next, we labeled Gas1^high^ SVZ NSCs from P55 to P60, and traced their fate during remyelination post CPZ or vehicle treatment (Fig 7A). In vehicle-treated mice, we observed a small number of Olig2^+ ^tdT^+^ cells in the CC (0.72 ± 0.09 cells/section), representing basal oligodendrogenesis from Gas1^high^ NSCs (Fig 7A and 7D). In contrast, 4 weeks post-CPZ treatment, we observed a marked increase of Olig2^+ ^tdT^+^ cells in the CC (5.83 ± 1.27 cells per section). Ten weeks post CPZ treatment, there is a further increase of Olig2^+ ^tdT^+^ cells (28.80 ± 6.52 cells/section) (Fig 7A and 7D), indicating that Gas1^high^ SVZ NSCs continue to generate oligodendrocyte lineage cells for a prolonged period of time during remyelination. From 4 to 10 weeks post-CPZ treatment, we also observed an increased number of total tdT^+^ cells and increased percentage of Olig2^+ ^tdT^+^ cells in the CC (Fig 7D), indicating continuous recruitment and differentiation of stem cells. At 10 weeks, ~ 80% of tdT^+^ cells in the CC are Olig2^+ ^, and ~ 60% of tdT^+^ cells express mature oligodendrocyte marker CNPase, exhibiting tdT-labeled myelinating processes colocalizing with MBP (Fig 7B, 7C, and 7E). These data collectively demonstrate that Gas1^high^ adult NSCs actively contribute to myelin repair after CPZ-induced demyelination.

## Discussion

Elucidating the developmental origin and functional heterogeneity of adult NSCs provides important insights into neurogenesis and gliogenesis in normal, aged, and disease conditions. In this study, we identified a subpopulation of dorsally-derived, Gas1^high^ adult NSCs with increased gliogenic potential, which plays an important role in adult oligodendrogenesis and myelin repair.

Gas1 is dynamically expressed during the transition from embryonic RG into postnatal and adult NSCs in the SVZ. In the embryonic VZ/SVZ, Gas1 is highly expressed in the dorsal RG cells and plays an important role in regulating their transition into postnatal NSCs. Postnatally, Gas1 continues to be expressed in NSCs at a reduced cellular proportion, delineating a subpopulation of qNSCs in the dorsal SVZ of adult and aged brains. The dynamics and specificity of Gas1 are different from other reported qNSC markers such as Vcam1, Lrig1, and Pdgfrβ [3,21,31], which also maintains NSCs in a quiescent state. Vcam1 is highly expressed in both astrocytes and qNSCs, while Lrig1 and Pdgfrβ label almost the entire qNSC population [3,21,31].

Compared to Gas1^low^ or Gas1^−^ NSCs, Gas1^high^ NSCs exhibit increased gliogenic potential. Remarkably, in aged brains Gas1^high^ NSCs continue to generate neuron and oligodendrocytes. Thus, high expression of Gas1 consistently marks multipotent NSCs during adulthood and aging. The efficiency of direct OPC differentiation from IPSCs/ESCs or NSCs is generally low or requires transduction of multiple transcription factors [32], which is a bottleneck of cell-based therapies for demyelinating diseases. Given Gas1 is membrane-bound cell surface marker, it could be easily used to sort out the pro-gliogenic NSC subpopulation for subsequent directed differentiation into oligodendrocyte lineage cells, which could potentially improve the efficiency of stem cell-based therapies.

Although Gas1 is a co-receptor for Patched1, Gas1^high^ cells normally do not appear to activate SHH signaling, expressing minimal Gli1. It is likely due to the fact that cells in the dorsal SVZ are not exposed to high-level SHH in the adult brain, in contrast to those in the ventral SVZ [18]. Under physiological conditions, Gas1 mainly functions as a cell cycle regulator and gatekeeper that maintains the NSCs in a relatively quiescent state. Nevertheless, Gas1^high^ cells retain the capacity to be activated by external SHH or SHH released upon demyelination [15], and can be mobilized to generate OPCs and oligodendrocytes in the CC. Thus, at the molecular level, Gas1 appears to hit two birds with one stone. Consequently, Gas1^high^ NSCs can serve as a reservoir for myelin repair in the adult and aged brain (Fig 7F). Interestingly, quiescent hair follicle stem cells expressing high levels of Gas1 can also sense Shh stimuli to promote regeneration [33], indicating it’s a conserved mechanism across different types of stem cells.

In summary, our study uncovers a subpopulation of long-term multipotent adult NSCs and defines their developmental origin, providing insights on the functional heterogeneity and plasticity of adult NSCs in health, aging, and disease, with implications for stem cell-based therapy.

### Experimental models and methods

#### Animals and ethics.

The *Gas1* CreER KI mouse was generated through genome editing following an adapted protocol [34] (Biocytogen, Beijing, China). We designed eight single guide RNAs (sgRNAs) and chose one efficiently targeting *Gas1* exon 1. The donor plasmid contained the iCreER^T^2-WPRE-polyA sequence inserted into right before the coding region of exon 1 to create a KI allele. Positive F0 mice were crossed to wild-type C57BL/6 mice to generate F1 founders, confirmed by PCR and Southern blot. The Ai9 Rosa-CAG-LSL-tdTomato reporter mouse was purchased from Jackson Laboratory (Strain 007909). For genotyping, tail DNA was extracted and amplified by PCR. The sequences for sgRNA and PCR primers are listed in S4 Table.

The mice were maintained on the C57BL/6 genetic background. They were housed in specific pathogen-free, temperature- and humidity-controlled rooms with a 12-h light/12-h dark cycle at the Experimental Animal Center of Sichuan University. All animal protocols have been approved by the Animal Care and Use Committee of Sichuan University, adhering to the National Guideline for Ethic Review of Animal Welfare, China (GB/T 35892-2018). Sex was not considered in the study design as it did not have a major role in our experiments.

#### Southern blot analysis.

Tail DNA from F1 *Gas1* CreER/+ KI mouse was extracted, digested using StuI and BspHI, separated on a 0.8% agarose gel and transferred to a nylon membrane. Gas1 exon 1 and iCreERT2-WPRE-polyA sequence specifically produces 6.7-kb, 5.4-kb and 3.9-kb DNA fragments detectable with the 5′ probe and WPRE probe, respectively. Southern blot probe sequences are listed in S5 Table.

#### Tamoxifen administration on *Gas1* CreER/+; RT/+ mice.

Tmx administration was performed as previously described [35,36]. Tmx (Sigma-Aldrich, T5648) was dissolved in corn oil (MCE, HY-Y1888) at a concentration of 20 mg/ml, sealed with foil to store at −20 °C, and pre-warmed in 37 °C water bath before use. For postnatal induction, mice were administered with Tmx through intraperitoneal injection at a daily dosage of 5 mg per 40 g body weight. For embryonic induction, pregnant females were injected intraperitoneally with a single dose of Tmx (50 μg/g body weight). The embryonic stage was determined by timed breeding.

#### Tissue preparation for histology.

Brain tissues were prepared for histological analysis according to a previous study [37]. Mice were perfused with phosphate-buffered saline (1× PBS) followed by 4% paraformaldehyde (PFA) (Sigma-Aldrich). The brains were dissected, placed in 4% PFA and fixed overnight at 4 °C, and then transferred to 30% sucrose at 4 °C for dehydration. The dehydrated brains were prepared as coronal or sagittal brain slices, embedded in O.C.T. compound (Tissue-Tek) and snap-frozen on dry ice. Serial sections were prepared at 10 µm thickness on a cryostat sectioning machine (Leica) and stored at −80 °C.

#### Immunofluorescence (IF).

IF on frozen sections was performed as previously described [37]. Frozen brain sections were dried in an oven at 37 °C for 30 min, rinsed and rehydrated with PBS, and treated with 0.3% Triton X-100 in PBS for 20 min at room temperature. For BrdU IF staining, the sections were additionally incubated in 2N HCl for 30 min and neutralized in 0.1 M sodium borate for 10 min at 37 °C [35]. Sections were then blocked with 2% goat serum in PBS for 1 h at room temperature, and incubated with primary antibodies overnight at 4 °C. Primary antibodies were visualized by species-specific goat secondary antibodies conjugated to Alexa Fluor dyes (Alexa 488/555/647, 1:500, Invitrogen). Sections were then stained with DAPI (1 μg/ml) for 5 min. Slides were coverslipped and imaged under an Olympus BX51 fluorescent microscope. Antibodies used in this study were: Gas1 (1:200, Donkey, R&D, AF2644), Living Colors DsRed Polyclonal Antibody (1:500, Rabbit, Takara, 632496), Olig2 (1:1000, Rabbit, Millipore, ab9610), CNPase (1:200, Mouse, Proteintech, 66729), GFAP (1:2000, Chicken, Abcam,ab4674), Nestin (1:100, Mouse, Abcam, ab6142), Foxj1 (1:500, Mouse, Invitrogen, 14-9965-82), Doublecortin (1:1000, Rabbit, Abcam, ab18723), NeuN (1:1000, Mouse, Abcam, ab104224), Sox2 (1:500, Rabbit, Abcam, ab92494), MBP (1:500, Rat, Millipore, mab386), Ki67 (1:500, Mouse, BD, 550609), BrdU (1:500, Rat, Abcam, ab6326).

For IF staining of adherently cultured cells, cells were fixed with 4% PFA for 15 min. After three washes with PBS, cells were treated with 0.2% Triton X-100 for 15 min, blocked with 5% goat serum in PBS for 1 hour at room temperature, and incubated with primary antibodies Tuj1 (1:2000, Mouse, Abcam, ab18207) and NG2 (1:500, Rabbit, Millipore, AB5320) overnight at 4 °C. Primary antibodies were visualized by species-specific goat secondary antibodies conjugated to Alexa Fluor dyes (Alexa 488/555/647, 1:1000, Invitrogen), and the nuclei were stained with DAPI (1 μg/ml). Stained cells were coverslipped and imaged under the Olympus BX51 fluorescent microscope. IF images presented in the figures are representative of at least three biological replicates in each group.

#### Neurosphere culture.

Mouse brains were cut into 1-mm slices and placed into PBS. SVZ tissues were carefully dissected under an anatomical microscope (Olympus), minced into small pieces, and incubated with 1 mg/ml collagenase type I (Gibco, 17100017) plus 0.5 mg/ml collagenase type IV (Gibco, 17104019) at 37 °C for 15 min, followed by mechanical dissociation through pipetting for 10 times. Dissociated cells were filtered through a 70 μm strainer (Corning, 352350), and centrifuged at 300*g*, 4 °C for 5 min. The pellet was resuspended with the NSCs culture medium (20 ng/ml bFGF (Gibco, 13256029), 20 ng/ml EGF (Gibco, PMG8041), 1× B27 (Gibco, 17504044), 1× N2 (Gibco, 17502048), 100 U/ml streptomycin and 100 mg/ml penicillin (Gibco, 15140122) in DMEM/F12 (Gibco, 11320033)). The cells were cultured in a 6-well ultra-low binding plate (Corning, 3471) for 7–10 days until neurosphere formation.

To passage NSCs, neurospheres were picked up by pipette and digested into single cells by Accutase (Gibco, A1110501) in 37 °C water bath for 5 min, with gentle pipetting. The digestion was terminated with DPBS (Gibco, C14190500BT). After centrifugation at 300*g* for 5 min, the pellet was resuspended with the NSCs culture medium and cultured in a 6-well ultra-low binding plate for 7–10 days until neurosphere formation.

#### Magnetic-activated cell sorting.

Neurospheres were dissociated into single cells and labeled with Gas1 primary antibody (1:200, Rabbit, Proteintech, 17903-1-AP) at 4 °C for 1 h, and labeled by Anti-Rabbit IgG magnetic microbeads (Miltenyi Biotec, 130-048-602) at 4 °C for 30 min. The cell suspension was loaded onto a MACS Column placed in the magnetic field of a MACS Separator, to retain magnetically labeled Gas1^+^ cells in the column while collecting unlabeled Gas1^−^ cells. Gas1^+^ cells were eluted and collected after removing the column from the magnetic field.

The Gas1^−^ and Gas1^+^ NSCs obtained by MACS were cultured separately with a complete medium preheated at 37 °C for 1 h. After 300*g* centrifugation, the fresh complete medium was resuspended and then cultured for 1 h. Repeat this process 5−6 times, and collect the cells labeled with Gas1 primary antibody (1:200, Rabbit, Proteintech, 17903-1-AP) at 4 °C for 1 h, and labeled by secondary antibodies conjugated to Alexa Fluor dyes (1:1000, Invitrogen) at 4 °C for 30 min. NSCs were stained with viability marker fixable viability stain 510 (FVS510, BD Horizon, 564406), and subjected to flow cytometry (Thermo Fisher, Attune NxT Acoustic Focusing Cytometer). Our purpose is to analyze the proportion of Gas1^+^ cells in the FVS510^ −^ population.

#### NSC differentiation.

Nunc Lab-Tek II chamber slides (ThermoFisher, 154534PK) were pre-coated with 0.01% Poly-d-Lysine Solution (Gibco, A3890401) and 5 µ g/ml of Laminin (Gibco, 23017015) in DMEM/F12 medium overnight at 37 °C, and wash with DPBS 2–3 times before use. 200–300 µm neurospheres were dissociated with Accutase and cultured on chamber slides in the NSC differentiation medium (1× B27, 1× N2, 100 U/ml streptomycin and 100 mg/ml penicillin in DMEM/F12) for 10–12 days. The medium was refreshed every 2 days.

#### Plasmids.

To generate the pLentiV2T-Gas1 CDS overexpressing plasmid, *Cas9* sequence in pLentiCRISPR V2 (pLentiV2T) vector (Addgene) was substituted with *Gas1* CDS sequence (ENSMUST00000065086.6), synthesized by Sangon Biotech. The plasmid sequence was confirmed by Sanger sequencing (Tsingke).

#### Lentiviral packaging and viral transfection.

Lentiviral packaging was performed according to a previous study [38]. Lentiviruses were produced in 293T cells using a calcium phosphate precipitation packaging system and concentrated (100×) using the Lenti-X Concentrator kit (Takara, 631232). When mouse NSCs reached 70% confluence 18 h after being plated in 6-well plates, 20 μl of concentrated lentivirus and 1 μl of polybrene (10 mg/ml) per well were added to the medium for 12 h. Infected mouse NSCs were then cultured in fresh medium for 48 h, followed by screening with puromycin (5 μg/ml) to establish stable pLentiV2T-Gas1 CDS overexpressing mouse NSCs for subsequent cell cycle analysis.

#### Cell cycle analysis.

We used the BeyoClick EdU Cell Proliferation Kit with Alexa Fluor 555 (Beyotime, C0075S) for this experiment. Neurospheres or SVZ tissue from mice were digested into a single cell, add 10 μM EdU (Beyotime, ST067) to culture at 37 °C for 20 h, and then fixed with Immunol Staining Fix Solution (Beyotime, P0098) for 15 min at room temperature. Then the cells were treated with Enhanced Immunostaining Permeabilization Buffer (Beyotime, P0097) for 15 min, incubated with EdU reaction solution at room temperature and avoid light for 30 min, and stained with DAPI (Solarbio, C0060) for 30 min and subsequently analyzed by Attune NxT Acoustic Focusing Cytometer (Thermo Fisher) The flow cytometry data was processed by FlowJo (BD, v10.9.0).

#### Reverse transcription-qPCR detect Gli1 expression.

The SVZ of 18 wild-type mice were carefully dissected and pooled, dissociated into single cells, and labeled with Gas1 primary antibody and magnetic microbeads. MACS were then performed on the cell suspension to precisely isolate the Gas1^−^ and Gas1^+^ cells, which were separately cultured in vitro. Immediately added 500 ng/ml of human SHH protein (MCE, HY-P70467) into the Gas1^−^ and Gas1^+^ cells (control group, without SHH). After cultured 48 h, the total mRNA was extracted from each group using the suitable EZ-press RNA Purification Kit (EZB, B0004DP). Subsequently, the extracted RNA was reverse transcribed into cDNA with the ExonScript RT SuperMix with dsDNase Kit (EXONGEN, A502-01) following the manufacturer’s instructions. Finally, RT-qPCR was employed to quantitatively analyze the expression levels of Gli1. The primers used in RT-qPCR are listed in S6 Table.

#### Injection of SHH into the LV.

Human SHH was injected into the LV according to previous studies [14,15]. Four-week-old *Gas1* CreER/ + ; RT/ + mice were induced with Tmx for 5 days at P26–P30. Eight microliter (3 μg) human SHH (R&D, 1845-SH/CF-25 μg) was then injected into the right LV at the following stereotaxic coordinates (to the bregma): anteroposterior + 0.2 mm, lateral + 0.8 mm, dorsoventral − 2.5 mm. Samples were collected for analysis 48 h after injection.

#### BrdU pulse-chase assay.

*Gas1* CreER/ + ; RT/ + mice were fed with BrdU in fresh drinking water (0.8 mg/ml) for 7 or 10 days, and chased for 3–5 weeks (specified in figure legends).

#### CPZ treatment and luxol fast blue staining.

CPZ-induced demyelination was performed according to a previous study [17]. After Tmx induction for 5 days, 8-week-old *Gas1* CreER/+; RT/+ mice were fed with 0.4% CPZ diet for 6 weeks to induce demyelination, and allowed to recover for 4 or 10 weeks. Luxol fast blue staining for myelin on tissue sections was performed according to the protocol from Luxol Fast Blue Stain Kit (Abcam, ab150675).

### scRNA-seq and analysis

#### Sample preparation.

In-house data: *Gas1*-CreER + , RT/ + mice were 5× Tmx induced from P26 to P30, and the mice were harvested at around 21 months of age. SVZ tissues from 8 mice were dissected and dissociated similar to the neurosphere culture protocol. Myelin was removed by Myelin Removal Beads (Miltenyi Biotec, 130-096-433). The SVZ cells were pooled for scRNA-seq at Novogene (Beijing, China). Over 10,000 viable cells were used for subsequent library construction and RNA sequencing.

Public data: GSE152281 dataset [8] was retrieved from the NCBI repository and processed using Cell Ranger to construct a gene expression matrix for subsequent analysis. GSE165554 dataset [9] was downloaded from GEO dataset, and we used the processed data directly.

#### Library preparation, sequencing, and alignment.

We followed the guidance of the 10× Genomics protocol to prepare single-cell suspensions using the Chromium Single-Cell Gene Expression Solution. Subsequently, RNA from single cells was barcoded, reverse transcribed, and amplified. The libraries constructed were sequenced using Illumina Novaseq 6000 platform by Novogene. Cell Ranger (v7.1.0) was used to generate the gene expression matrix, employing default settings based on the mm10 mouse reference genome, which includes annotations for exogenous genes such as the tdTomato and *Gas1*-CreET sequences. Gene expression was quantified based on the unique molecular identifier. Cells were later excluded for downstream analysis if they exhibited more than 10% mitochondrial gene proportions or had fewer than 200 gene counts. For GSE152281, the quality control of the expression matrix strictly followed the methodology described by Borrett and colleagues.

### scRNA-seq data analysis pipeline

We utilized Seurat (v4.4.0) [39] for data normalization (NormalizeData, LogNormalize method, scaling factor 10,000), scaling of data features (ScaleData), and detection of variable genes (FindVariableGenes with vst method). Principal component analysis (PCA) was performed on these variable genes with RunPCA. The statistically significant principal components were used to generate a two-dimensional UMAP. Cell clustering was conducted using the original Louvain algorithm (FindClusters). Differential gene expression analysis was performed using the FindAllMarkers function in Seurat with default settings, identifying significant genes with a log2 fold change greater than 1 and an adjusted *p*-value below 0.01. We conduct statistical tests in single-cell analysis using the rstatix package (v0.7.2) and the stat_compare_means function from the ggpubr package (v0.6.0). For differential gene expression analysis, we employed the FindMarker function with default parameters. Differential genes with an absolute log2 fold change greater than 0.25 and a *P*-value less than 0.05 were selected for GO enrichment analysis. The enrichment analysis was performed using the enrichGO function from the clusterProfiler package (v4.4.4) [40], and pathways with *p*-values greater than 0.05 were filtered out.

### Trajectory inference and pseudotime ordering

The single-cell pseudotime trajectory was constructed using a combination of Spectral Embedding [41] and Principal Curve [42]. Briefly, the expression profiles of single NSCs were extracted from the Borrett and colleagues dataset following their original cell-type assignment (E14.5/E17.5: RPs, P2/P20/P34/P61: NSCs, P6: qNSCs). These NSC data were merged and normalized (NormalizeData, normalization. method =  LogNormalize, while others were set to default values) and subjected to variable gene selection (FindVariableFeatures, while others were set to default values) in Seurat. The expression matrix of highly variable genes was used as the input for Spectral Embedding. And principal curve fitting was then applied to the dimensionality-reduced results to obtain a fitted time curve. Based on the position along the fitted curve, the pseudotime progression of individual cells along the trajectory can be inferred. The differential analysis of pseudo-temporal data was conducted using the runPseudotimeDE function from the PseudotimeDE package [22] (model = ‘nb’, while others were set to default values), resulting in a list of differentially expressed genes. Differentially expressed genes were identified based on their degrees of variation and significance. Subsequently, gene expression and pseudo-temporal profiles were fitted using a generalized linear model (generated by runPseudotimeDE), and pseudotime curves of gene expression were plotted by ggplot (v3.5.0) [43].

### Quantification and statistical analysis

Original statistical data are provided as S1 Data in the supplementary information. No statistical method was used to predetermine sample size. The data distribution was assumed to be normal, but this was not formally tested. We excluded single-cell transcriptomes that failed quality control (described in the ‘Library preparation, sequencing, and alignment’ section). All animals and cultured cells were randomly assigned to each group. Multiple anatomically comparable sections, ~ 100 μm apart, were imaged for quantification (see S1 Data for details). The cell number and colocalization were quantified using the ImageJ software, and compared by unpaired Student *t* test using GraphPad Prism 8 software. Wilcoxon test was used to compare differential gene expression in scRNA-seq analysis. Highly variable genes along the pseudotime trajectory were determined using the Chi-Square test. *P*-values or adjusted *P*-values < 0.05 were considered statistically significant.

## Supporting information

S1 Fig Cell-type annotation of single cells from various embryonic and early developmental time points in Borrett and colleagues datasets. UMAP plots of major cell types in Borrett and colleagues datasets, including E14.5 (*n* =  8,096 cells), E17.5 (*n* =  1,846 cells), P2 (*n* =  2,019 cells), P6–7 (*n* =  7,972 cells), P20 (*n* =  1,943 cells), P34 (*n* =  2,135 cells), along with dot plots showing the expression level and percentage of marker genes in each cell type.
(PDF)

S2 Fig Cell-type annotation of single cells from various adult developmental time points in Borrett and colleagues and Silla and colleagues datasets. **(A)** UMAP plots of major cell types in Borrett and colleagues datasets, including P61 (*n* =  2,361 cells), along with dot plots showing the expression level and percentage of marker genes in each cell type. **(B)** UMAP plots of major cell types in Silla and colleagues dataset (*n* =  24,261 cells), along with dot plots showing the expression level and percentage of marker genes in each cell type. **(C, D)** Scatter plots showing the expression of Fabp7, Mki67, Top2a, Gmnn, Cdkn1c, and Vcam1 in NSCs along the pseudotime trajectory.
(PDF)

S3 Fig Validation of the Gas1 *CreER* knock-in allele. **(A)** Genotyping of F1 Gas1 *CreER*/+ and WT mice using PCR primers F1, R1, R2. *Gas1* wild-type band (F1, R1): 726 bp. *Gas1* CreER KI band (F1, R2): 417 bp. The original gel images can be found in S1 Raw Images. **(B)** Southern blot using the tail DNA of F1 *Gas1* CreER/+ and WT mice. After digestion by StuI (upper panel) or BspHI (lower panel) restriction enzymes, 5′ probe identifies wild-type (6.7 kb) and KI bands (5.4 kb), and WPRE probe identifies KI-specific bands (3.9 kb). **(C)** *Gas1* CreER/+ ; RT/+ mice were Tmx-induced from P26 to P30 and analyzed at P31 using sagittal brain sections. Representative IF co-labeling of Gas1 and tdT in the dorsal anterior SVZ at P31 are shown. Arrows highlight examples of Gas1^+^tdT^+^ NSCs. **(D)** *Gas1* CreER/+ ; RT/+ mice were Tmx-induced at E14.5 and analyzed at P21 using sagittal brain sections. Representative IF co-labeling of Foxj1 and tdT (DsRed) in the P21 SVZ are shown. Arrows highlight colocalizing cells and arrowhead labels non-colocalizing cells. Quantification of the percentage of NSCs or ECs among total tdT^+^ cells in the SVZ. *n* =  2 mice. LV, lateral ventricles; RMS, rostral migratory stream; CC, corpus callosum; SVZ, subventricular zone; OB, olfactory bulb; ST, striatum. Scale bars, 100 μm.
(PDF)

S4 Fig P8-labeled Gas1^high^ cells give rise to P30 SVZ NSCs and undergo multi-lineage differentiation. **(A)** Gas1 *CreER*/+ ; RT/+ mice were Tmx-induced at P8 (125 μg/g body weight) and analyzed at P30 using sagittal brain sections. Representative IF co-labeling of GFAP, tdT (DsRed) and Nestin in the P30 SVZ are shown. Arrows highlight examples of GFAP^+^tdT^+^Nestin^+^ cells. **(B)** Representative IF co-labeling of tdT and NeuN in the RMS or OB at P30, respectively. Arrows highlight examples of colocalizing cells. **(C)** Representative IF co-labeling of tdT and Olig2 in the CC at P30. Arrows highlight examples of colocalizing cells. **(D)** Representative IF co-labeling of tdT and GFAP in the CC at P30. Arrows highlight examples of colocalizing cells. SVZ, subventricular zone; LV, lateral ventricles; CC, corpus callosum; RMS, rostral migratory stream; OB, olfactory bulb; Scale bars, 100 μm. The original data underlying S4A Fig can be found in S1 Data.
(PDF)

S5 Fig Gas1 is a gatekeeper of quiescence maintenance in adult NSCs. **(A)** Representative flow cytometric results of MACS-Gas1 + cells and MACS-Gas1- cells. Isotype control as a negative control. **(B)** Quantification of the percentage of stained-Gas1^+^ cells among MACS-Gas1^−^ cells and MACS-Gas1^+^ cells. *n* =  3 for each group. Data represent Mean ±  SD. **(C)** Representative flow cytometry charts in the cell cycle assay comparing cultured NSCs infected with Gas1-V2TH or Gas1-OE lentiviruses stained with EdU and DAPI. The proportions of cells at different phases of the cell cycle (G0/1, S and G2/M) are indicated. **(D)** Quantification of the percentage of cells at different phases of the cell cycle from Gas1-V2TH and Gas1-OE NSCs in (C). *n* =  3 biological replicates. Data represent Mean ±  SD. **(E)** Representative flow cytometry charts in the cell cycle assay comparing cultured NSCs from control or Gas1-KO mice SVZ stained with EdU and DAPI. The proportions of cells at different phases of the cell cycle (G0/1, S and G2/M) are indicated. **(F)** Quantification of the percentage of cells at different phases of the cell cycle from control and Gas1-KO NSCs in (E). *n* =  3 biological replicates. Data represent Mean ±  SD. **(G)** Pseudotime ordering of NSCs lineage development in adult SVZ datasets and scatter plots showing the expression of Gas1 and Mki67 along the pseudotime trajectory. Top, Silla and colleagues dataset (*n* =  14,660 cells). Bottom, Borrett and colleagues dataset (*n* =  3,045 cells). The original data underlying S5B, S5D, and S5F Fig can be found in S1 Data.
(PDF)

S6 Fig Validation of CPZ-induced demyelination and remyelination after CPZ removal. **(A)** Gas1 *CreER*/+ ; RT/+ mice at P60 were fed with normal (No CPZ) or 0.2% CPZ (6w CPZ) diet from P60 to P102 and analyzed at P103. Representative images of Luxol fast blue staining on corona brain sections from No CPZ and 6w CPZ groups are shown. Scale bar, 200 μm. **(B)** *Gas1* CreER/+; RT/+ mice were fed with normal (No CPZ) or 0.2% CPZ in diet from P60 to P102, allowed to recover and analyzed at P130 (4 weeks post-CPZ) or P172 (10 weeks post-CPZ). Coronal brain sections from No CPZ, 4 and 10 weeks post-CPZ groups were IF stained for MBP, Olig2 or GFAP. Scale bars, 100 μm. LV, lateral ventricles; CC, corpus callosum; CPZ, cuprizone.
(PDF)

S1 Table Borrett and colleagues E14.5-P61 NSCS data pseudotime differential analysis gene.
(XLSX)

S2 Table Overlap genes of dynamic genes at multiple time points in S1 Table and adult SVZ NSC marker genes.
(XLSX)

S3 Table Cell-type specific gene expression in the single-cell transcriptomic analysis by house data.
(XLSX)

S4 Table The sequences for sgRNA and PCR primers. (XLSX)

S5 Table Southern blot details.
(XLSX)

S6 Table List of primers used for RT-qPCR for mRNAs.
(XLSX)

S1 Data Numerical data supporting figures.
(XLSX)

S1 Raw Images The original blot and gel images.
(PDF)

## Abbreviations

aNSCs: active neural stem cells;
CC: corpus callosum
CPZ: cuprizone
CTX: cortex
EPCs: ependymal cells
IF: immunofluorescence
KI: knock-in
LGE: lateral ganglionic eminence
LV: lateral ventricle
NBs: neuroblasts
NSCs: neural stem cells
OB: olfactory bulb
PBS: phosphate-buffered saline
PCA: principal component analysis
PFA: paraformaldehyde
qNSCs: quiescent neural stem cells
RG: radial glia
RMS: rostral migratory stream
scRNA-seq: single-cell RNA sequencing
sgRNAs: single guide RNAs
ST: striatum
SVZ: subventricular zone
TAPs: transit-amplifying progenitors
tdT^+^: tdTomato-positive
Tmx: tamoxifen
VZ: ventricular zone

## References

[1] Bond AM, Ming G-L, Song H. Ontogeny of adult neural stem cells in the mammalian brain. Curr Top Dev Biol. 2021;142:67–98. doi: 10.1016/bs.ctdb.2020.11.002 33706926 PMC8363052

[2] Furutachi S, Miya H, Watanabe T, Kawai H, Yamasaki N, Harada Y, et al. Slowly dividing neural progenitors are an embryonic origin of adult neural stem cells. Nat Neurosci. 2015;18(5):657–65. doi: 10.1038/nn.3989 25821910

[3] Hu X-L, Chen G, Zhang S, Zheng J, Wu J, Bai Q-R, et al. Persistent expression of VCAM1 in radial glial cells is required for the embryonic origin of postnatal neural stem cells. Neuron. 2017;95(2):309–325.e6. doi: 10.1016/j.neuron.2017.06.047 28728023

[4] Ortiz-Álvarez G, Daclin M, Shihavuddin A, Lansade P, Fortoul A, Faucourt M, et al. Adult neural stem cells and multiciliated ependymal cells share a common lineage regulated by the geminin family members. Neuron. 2019;102(1):159–172.e7. doi: 10.1016/j.neuron.2019.01.051 30824354 PMC6449116

[5] Redmond SA, Figueres-Oñate M, Obernier K, Nascimento MA, Parraguez JI, López-Mascaraque L, et al. Development of ependymal and postnatal neural stem cells and their origin from a common embryonic progenitor. Cell Rep. 2019;27(2):429–441.e3. doi: 10.1016/j.celrep.2019.01.088 30970247 PMC6499605

[6] Codega P, Silva-Vargas V, Paul A, Maldonado-Soto AR, Deleo AM, Pastrana E, et al. Prospective identification and purification of quiescent adult neural stem cells from their in vivo niche. Neuron. 2014;82(3):545–59. doi: 10.1016/j.neuron.2014.02.039 24811379 PMC4360885

[7] Llorens-Bobadilla E, Zhao S, Baser A, Saiz-Castro G, Zwadlo K, Martin-Villalba A. Single-cell transcriptomics reveals a population of dormant neural stem cells that become activated upon brain injury. Cell Stem Cell. 2015;17(3):329–40. doi: 10.1016/j.stem.2015.07.002 26235341

[8] Borrett MJ, Innes BT, Jeong D, Tahmasian N, Storer MA, Bader GD, et al. Single-cell profiling shows murine forebrain neural stem cells reacquire a developmental state when activated for adult neurogenesis. Cell Rep. 2020;32(6):108022. doi: 10.1016/j.celrep.2020.108022 32783944

[9] Cebrian-Silla A, Nascimento MA, Redmond SA, Mansky B, Wu D, Obernier K, et al. Single-cell analysis of the ventricular-subventricular zone reveals signatures of dorsal and ventral adult neurogenesis. Elife. 2021;10:e67436. doi: 10.7554/eLife.67436 34259628 PMC8443251

[10] Merkle FT, Mirzadeh Z, Alvarez-Buylla A. Mosaic organization of neural stem cells in the adult brain. Science. 2007;317(5836):381–4. doi: 10.1126/science.1144914 17615304

[11] Merkle FT, Fuentealba LC, Sanders TA, Magno L, Kessaris N, Alvarez-Buylla A. Adult neural stem cells in distinct microdomains generate previously unknown interneuron types. Nat Neurosci. 2014;17(2):207–14. doi: 10.1038/nn.3610 24362763 PMC4100623

[12] Menn B, Garcia-Verdugo JM, Yaschine C, Gonzalez-Perez O, Rowitch D, Alvarez-Buylla A. Origin of oligodendrocytes in the subventricular zone of the adult brain. J Neurosci. 2006;26(30):7907–18. doi: 10.1523/JNEUROSCI.1299-06.2006 16870736 PMC6674207

[13] Ortega F, Gascón S, Masserdotti G, Deshpande A, Simon C, Fischer J, et al. Oligodendrogliogenic and neurogenic adult subependymal zone neural stem cells constitute distinct lineages and exhibit differential responsiveness to Wnt signalling. Nat Cell Biol. 2013;15(6):602–13. doi: 10.1038/ncb2736 23644466

[14] Loulier K, Ruat M, Traiffort E. Increase of proliferating oligodendroglial progenitors in the adult mouse brain upon Sonic hedgehog delivery in the lateral ventricle. J Neurochem. 2006;98(2):530–42. doi: 10.1111/j.1471-4159.2006.03896.x 16805844

[15] Ferent J, Zimmer C, Durbec P, Ruat M, Traiffort E. Sonic hedgehog signaling is a positive oligodendrocyte regulator during demyelination. J Neurosci. 2013;33(5):1759–72. doi: 10.1523/JNEUROSCI.3334-12.2013 23365216 PMC6619133

[16] Ahn S, Joyner AL. In vivo analysis of quiescent adult neural stem cells responding to Sonic hedgehog. Nature. 2005;437(7060):894–7. doi: 10.1038/nature03994 16208373

[17] Samanta J, Grund EM, Silva HM, Lafaille JJ, Fishell G, Salzer JL. Inhibition of Gli1 mobilizes endogenous neural stem cells for remyelination. Nature. 2015;526(7573):448–52. doi: 10.1038/nature14957 26416758 PMC4970518

[18] Tong CK, Fuentealba LC, Shah JK, Lindquist RA, Ihrie RA, Guinto CD, et al. A Dorsal SHH-dependent domain in the V-SVZ produces large numbers of oligodendroglial lineage cells in the postnatal brain. Stem Cell Reports. 2015;5(4):461–70. doi: 10.1016/j.stemcr.2015.08.013 26411905 PMC4624995

[19] Machold R, Hayashi S, Rutlin M, Muzumdar MD, Nery S, Corbin JG, et al. Sonic hedgehog is required for progenitor cell maintenance in telencephalic stem cell niches. Neuron. 2003;39(6):937–50. doi: 10.1016/s0896-6273(03)00561-0 12971894

[20] Ihrie RA, Shah JK, Harwell CC, Levine JH, Guinto CD, Lezameta M, et al. Persistent sonic hedgehog signaling in adult brain determines neural stem cell positional identity. Neuron. 2011;71(2):250–62. doi: 10.1016/j.neuron.2011.05.018 21791285 PMC3346180

[21] Delgado AC, Maldonado-Soto AR, Silva-Vargas V, Mizrak D, von Känel T, Tan KR, et al. Release of stem cells from quiescence reveals gliogenic domains in the adult mouse brain. Science. 2021;372(6547):1205–9. doi: 10.1126/science.abg8467 34112692

[22] Song D, Li JJ. PseudotimeDE: inference of differential gene expression along cell pseudotime with well-calibrated p-values from single-cell RNA sequencing data. Genome Biol. 2021;22(1):124. doi: 10.1186/s13059-021-02341-y 33926517 PMC8082818

[23] Del Sal G, Ruaro ME, Philipson L, Schneider C. The growth arrest-specific gene, gas1, is involved in growth suppression. Cell. 1992;70(4):595–607. doi: 10.1016/0092-8674(92)90429-g 1505026

[24] Allen BL, Tenzen T, McMahon AP. The Hedgehog-binding proteins Gas1 and Cdo cooperate to positively regulate Shh signaling during mouse development. Genes Dev. 2007;21(10):1244–57. doi: 10.1101/gad.1543607 17504941 PMC1865495

[25] Martinelli DC, Fan C-M. Gas1 extends the range of Hedgehog action by facilitating its signaling. Genes Dev. 2007;21(10):1231–43. doi: 10.1101/gad.1546307 17504940 PMC1865494

[26] Madisen L, Zwingman TA, Sunkin SM, Oh SW, Zariwala HA, Gu H, et al. A robust and high-throughput Cre reporting and characterization system for the whole mouse brain. Nat Neurosci. 2010;13(1):133–40. doi: 10.1038/nn.2467 20023653 PMC2840225

[27] Gorski JA, Talley T, Qiu M, Puelles L, Rubenstein JLR, Jones KR. Cortical excitatory neurons and glia, but not GABAergic neurons, are produced in the Emx1-expressing lineage. J Neurosci. 2002;22(15):6309–14. doi: 10.1523/JNEUROSCI.22-15-06309.2002 12151506 PMC6758181

[28] Lee CS, May NR, Fan CM. Transdifferentiation of the ventral retinal pigmented epithelium to neural retina in the growth arrest specific gene 1 mutant. Dev Biol. 2001;236(1):17–29. doi: 10.1006/dbio.2001.0280 11456441

[29] Hagey DW, Muhr J. Sox2 acts in a dose-dependent fashion to regulate proliferation of cortical progenitors. Cell Rep. 2014;9(5):1908–20. doi: 10.1016/j.celrep.2014.11.013 25482558

[30] Matsushima GK, Morell P. The neurotoxicant, cuprizone, as a model to study demyelination and remyelination in the central nervous system. Brain Pathol. 2001;11(1):107–16. doi: 10.1111/j.1750-3639.2001.tb00385.x 11145196 PMC8098267

[31] Marqués-Torrejón MÁ, Williams CAC, Southgate B, Alfazema N, Clements MP, Garcia-Diaz C, et al. LRIG1 is a gatekeeper to exit from quiescence in adult neural stem cells. Nat Commun. 2021;12(1):2594. doi: 10.1038/s41467-021-22813-w 33972529 PMC8110534

[32] Ehrlich M, Mozafari S, Glatza M, Starost L, Velychko S, Hallmann A-L, et al. Rapid and efficient generation of oligodendrocytes from human induced pluripotent stem cells using transcription factors. Proc Natl Acad Sci U S A. 2017;114(11):E2243–52. doi: 10.1073/pnas.1614412114 28246330 PMC5358375

[33] Hsu Y-C, Li L, Fuchs E. Transit-amplifying cells orchestrate stem cell activity and tissue regeneration. Cell. 2014;157(4):935–49. doi: 10.1016/j.cell.2014.02.057 24813615 PMC4041217

[34] Wang H, Yang H, Shivalila CS, Dawlaty MM, Cheng AW, Zhang F, et al. One-step generation of mice carrying mutations in multiple genes by CRISPR/Cas-mediated genome engineering. Cell. 2013;153(4):910–8. doi: 10.1016/j.cell.2013.04.025 23643243 PMC3969854

[35] Wang Y, Kim E, Wang X, Novitch BG, Yoshikawa K, Chang L-S, et al. ERK inhibition rescues defects in fate specification of Nf1-deficient neural progenitors and brain abnormalities. Cell. 2012;150(4):816–30. doi: 10.1016/j.cell.2012.06.034 22901811 PMC3427010

[36] Xu H-T, Han Z, Gao P, He S, Li Z, Shi W, et al. Distinct lineage-dependent structural and functional organization of the hippocampus. Cell. 2014;157(7):1552–64. doi: 10.1016/j.cell.2014.03.067 24949968 PMC4120073

[37] Wang X, Zhou R, Xiong Y, Zhou L, Yan X, Wang M, et al. Sequential fate-switches in stem-like cells drive the tumorigenic trajectory from human neural stem cells to malignant glioma. Cell Res. 2021;31(6):684–702. doi: 10.1038/s41422-020-00451-z 33390587 PMC8169837

[38] Yao P, Xiao P, Huang Z, Tang M, Tang X, Yang G, et al. Protein-level mutant p53 reporters identify druggable rare precancerous clones in noncancerous tissues. Nat Cancer. 2023;4(8):1176–92. doi: 10.1038/s43018-023-00608-w 37537298

[39] Hao Y, Hao S, Andersen-Nissen E, Mauck WM 3rd, Zheng S, Butler A, et al. Integrated analysis of multimodal single-cell data. Cell. 2021;184(13):3573–3587.e29. doi: 10.1016/j.cell.2021.04.048 34062119 PMC8238499

[40] Yu G, Wang L-G, Han Y, He Q-Y. clusterProfiler: an R package for comparing biological themes among gene clusters. OMICS. 2012;16(5):284–7. doi: 10.1089/omi.2011.0118 22455463 PMC3339379

[41] Belkin M, Niyogi P. Laplacian eigenmaps for dimensionality reduction and data representation. Neural Comput. 2003;15(6):1373–96. doi: 10.1162/089976603321780317

[42] Hastie T, Stuetzle W. Principal curves. J Am Stat Assoc. 1989;84(406):502–16. doi: 10.1080/01621459.1989.10478797

[43] Wickham H. ggplot2: Elegant Graphics for Data Analysis. Springer-Verlag: New York; 2016.

