# Supplementary figures and images for: Gas1-high quiescent neural stem cells are multipotent and produce oligodendrocytes during aging and after demyelinating injury
Source: PLoS Biol. 2025 Apr 3;23(4):e3003100. doi: 10.1371/journal.pbio.3003100 (PMC11990765; doi:10.1371/journal.pbio.3003100)

**Figure S1**

Borrett et al. E14.5

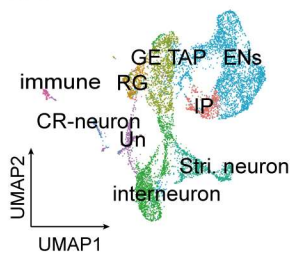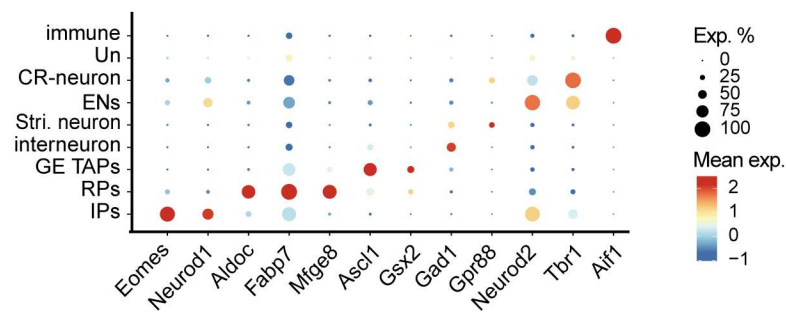

Borrett et al. E17.5

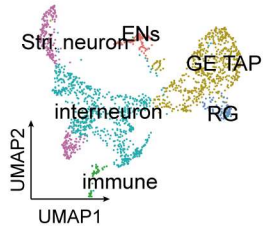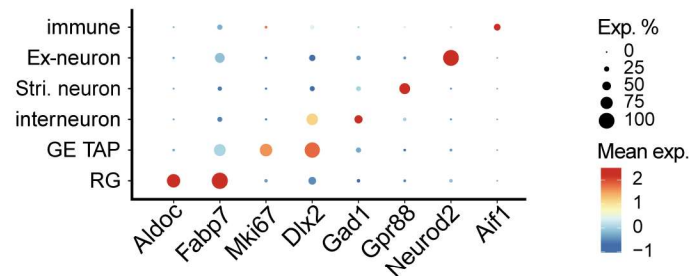

Borrett et al. P2

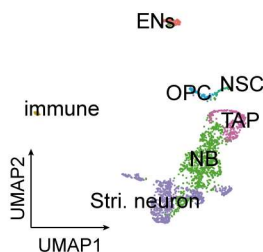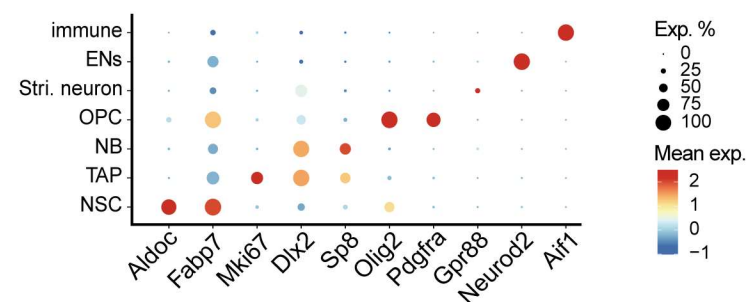

Borrett et al. P6/7

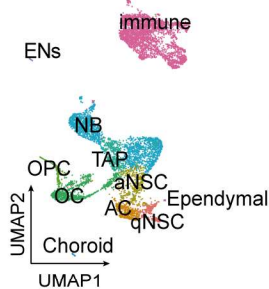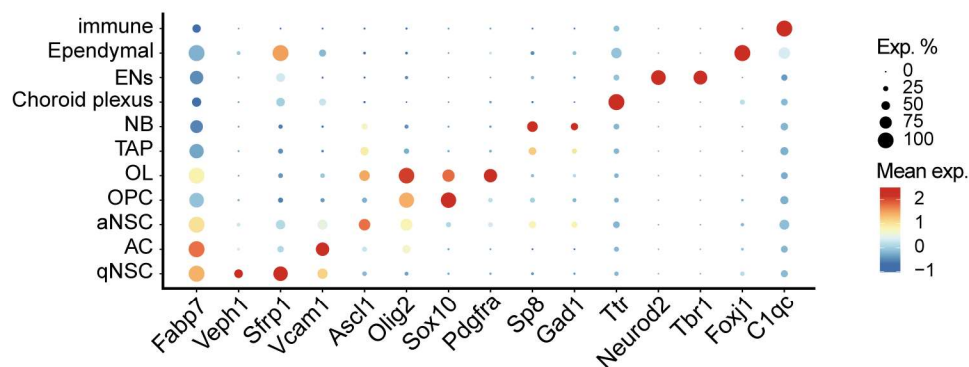

Borrett et al. P20

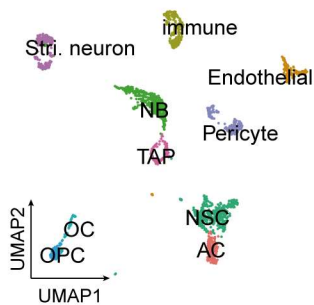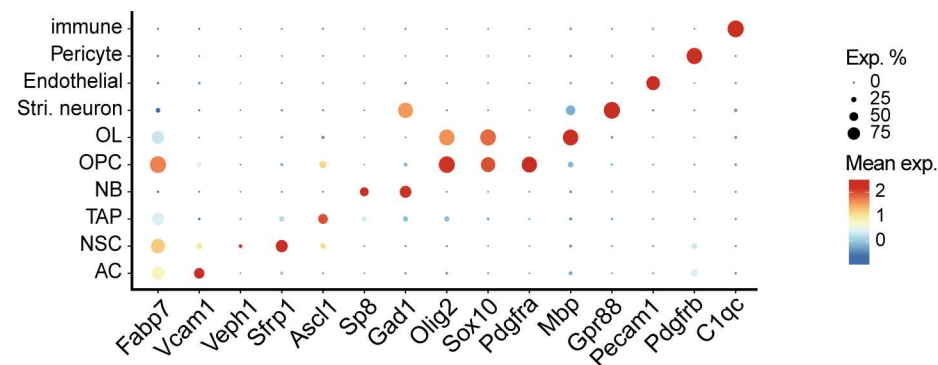

Borrett et al. P34

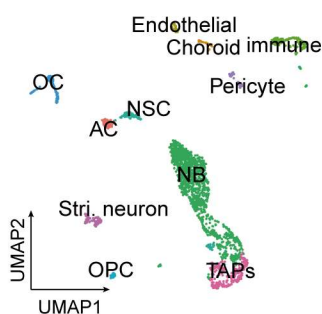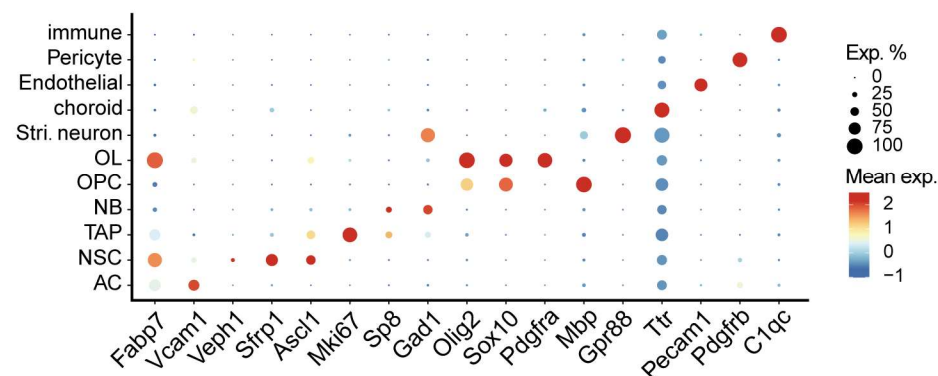

Supplement: S1 Fig — UMAP plots of major cell types in Borrett and colleagues datasets, including E14.5 (n = 8,096 cells), E17.5 (n = 1,846 cells), P2 (n = 2,019 cells), P6–7 (n = 7,972 cells), P20 (n = 1,943 cells), P34 (n = 2,135 cells), along with dot plots showing the expression level and percentage of marker genes in each cell type. (PDF) [file pbio.3003100.s001.pdf]

**Figure S2**

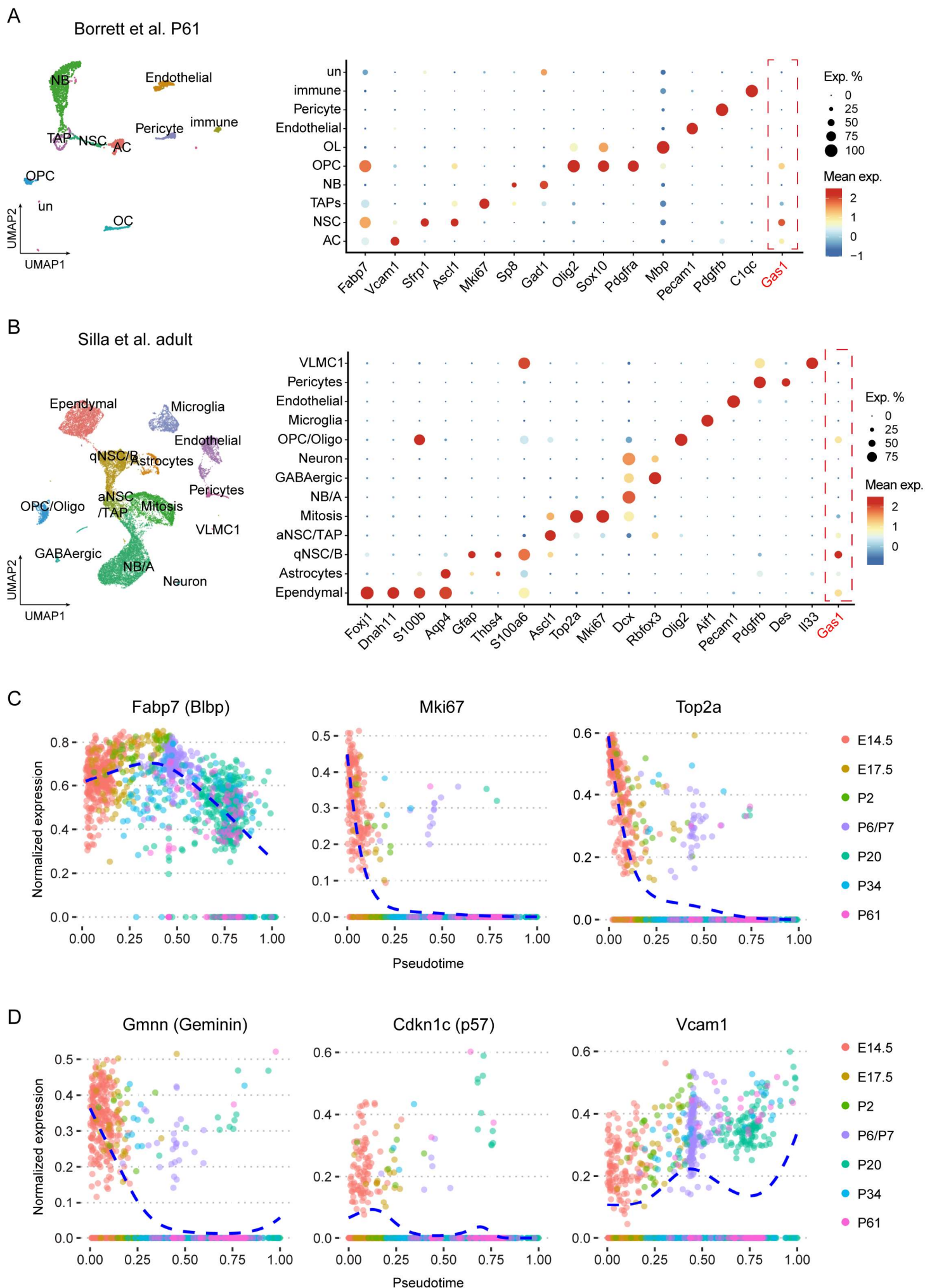

Supplement: S2 Fig — (A) UMAP plots of major cell types in Borrett and colleagues datasets, including P61 (n = 2,361 cells), along with dot plots showing the expression level and percentage of marker genes in each cell type. (B) UMAP plots of major cell types in Silla and colleagues dataset (n = 24,261 cells), along with dot plots showing the expression level and percentage of marker genes in each cell type. (C, D) Scatter plots showing the expression of Fabp7, Mki67, Top2a, Gmnn, Cdkn1c, and Vcam1 in NSCs along the pseudotime trajectory. (PDF) [file pbio.3003100.s002.pdf]

Figure S3

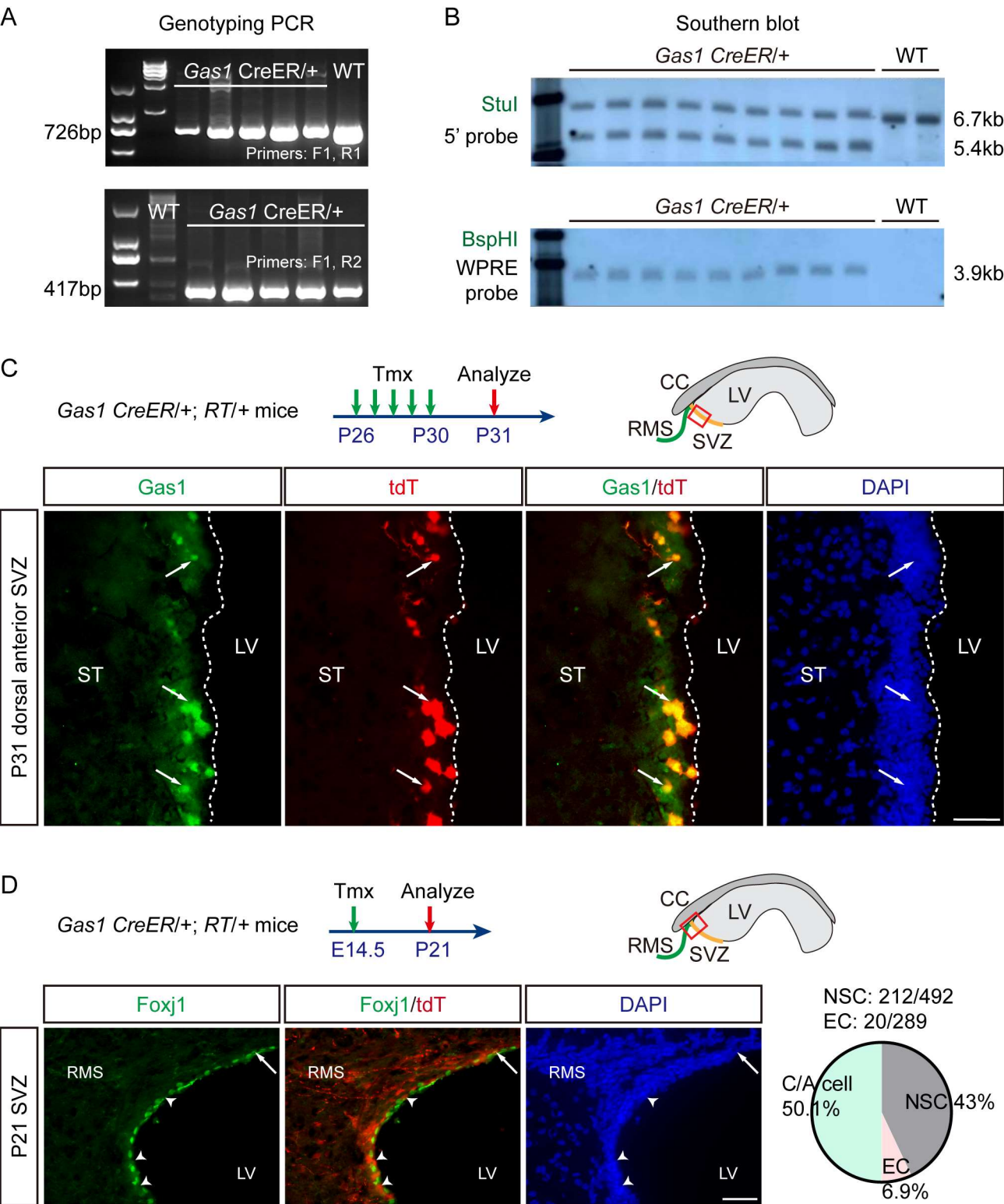

Supplement: S3 Fig — (A) Genotyping of F1 Gas1 CreER/+ and WT mice using PCR primers F1, R1, R2. Gas1 wild-type band (F1, R1): 726 bp. Gas1 CreER KI band (F1, R2): 417 bp. The original gel images can be found in S1 Raw Images. (B) Southern blot using the tail DNA of F1 Gas1 CreER/+ and WT mice. After digestion by StuI (upper panel) or BspHI (lower panel) restriction enzymes, 5′ probe identifies wild-type (6.7 kb) and KI bands (5.4 kb), and WPRE probe identifies KI-specific bands (3.9 kb). (C) Gas1 CreER/+ ; RT/+ mice were Tmx-induced from P26 to P30 and analyzed at P31 using sagittal brain sections. Representative IF co-labeling of Gas1 and tdT in the dorsal anterior SVZ at P31 are shown. Arrows highlight examples of Gas1+tdT+ NSCs. (D) Gas1 CreER/+ ; RT/+ mice were Tmx-induced at E14.5 and analyzed at P21 using sagittal brain sections. Representative IF co-labeling of Foxj1 and tdT (DsRed) in the P21 SVZ are shown. Arrows highlight colocalizing cells and arrowhead labels non-colocalizing cells. Quantification of the percentage of NSCs or ECs among total tdT+ cells in the SVZ. n = 2 mice. LV, lateral ventricles; RMS, rostral migratory stream; CC, corpus callosum; SVZ, subventricular zone; OB, olfactory bulb; ST, striatum. Scale bars, 100 μm. (PDF) [file pbio.3003100.s003.pdf]

Figure S4

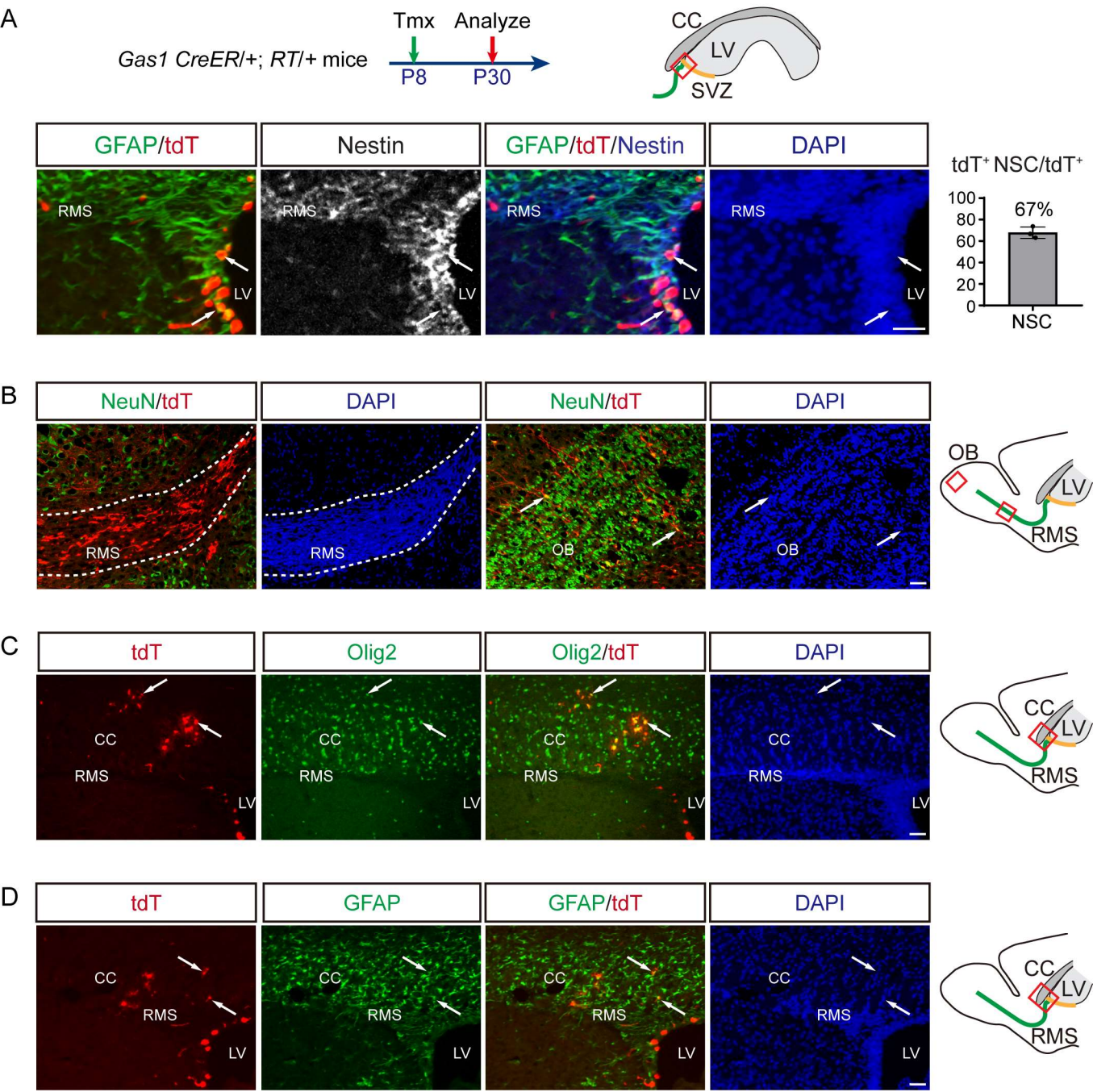

Supplement: S4 Fig — (A) Gas1 CreER/+ ; RT/+ mice were Tmx-induced at P8 (125 μg/g body weight) and analyzed at P30 using sagittal brain sections. Representative IF co-labeling of GFAP, tdT (DsRed) and Nestin in the P30 SVZ are shown. Arrows highlight examples of GFAP+tdT+Nestin+ cells. (B) Representative IF co-labeling of tdT and NeuN in the RMS or OB at P30, respectively. Arrows highlight examples of colocalizing cells. (C) Representative IF co-labeling of tdT and Olig2 in the CC at P30. Arrows highlight examples of colocalizing cells. (D) Representative IF co-labeling of tdT and GFAP in the CC at P30. Arrows highlight examples of colocalizing cells. SVZ, subventricular zone; LV, lateral ventricles; CC, corpus callosum; RMS, rostral migratory stream; OB, olfactory bulb; Scale bars, 100 μm. The original data underlying S4A Fig can be found in S1 Data. (PDF) [file pbio.3003100.s004.pdf]

**Figure S5**

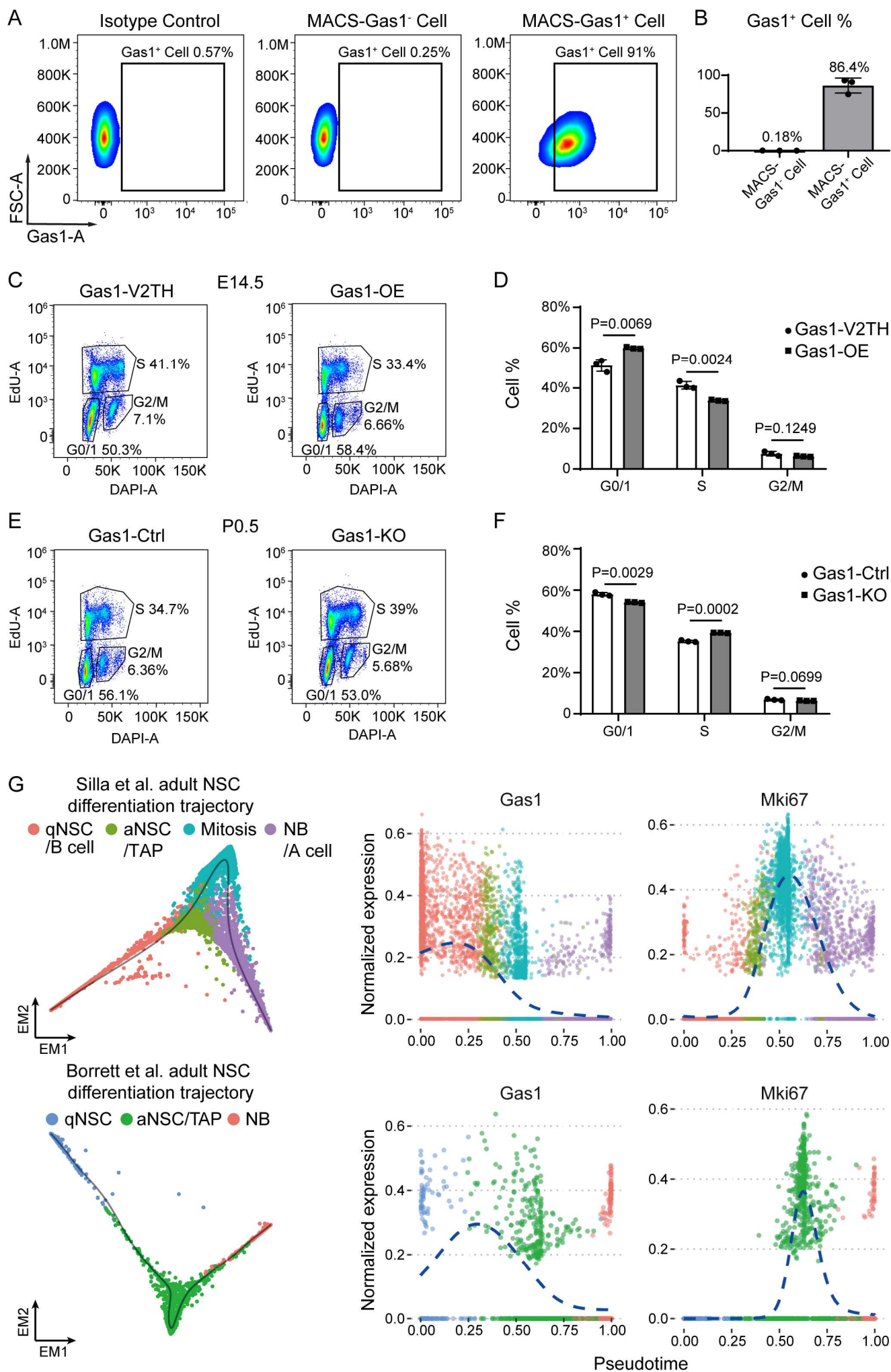

Supplement: S5 Fig — (A) Representative flow cytometric results of MACS-Gas1 + cells and MACS-Gas1- cells. Isotype control as a negative control. (B) Quantification of the percentage of stained-Gas1+ cells among MACS-Gas1− cells and MACS-Gas1+ cells. n = 3 for each group. Data represent Mean ± SD. (C) Representative flow cytometry charts in the cell cycle assay comparing cultured NSCs infected with Gas1-V2TH or Gas1-OE lentiviruses stained with EdU and DAPI. The proportions of cells at different phases of the cell cycle (G0/1, S and G2/M) are indicated. (D) Quantification of the percentage of cells at different phases of the cell cycle from Gas1-V2TH and Gas1-OE NSCs in (C). n = 3 biological replicates. Data represent Mean ± SD. (E) Representative flow cytometry charts in the cell cycle assay comparing cultured NSCs from control or Gas1-KO mice SVZ stained with EdU and DAPI. The proportions of cells at different phases of the cell cycle (G0/1, S and G2/M) are indicated. (F) Quantification of the percentage of cells at different phases of the cell cycle from control and Gas1-KO NSCs in (E). n = 3 biological replicates. Data represent Mean ± SD. (G) Pseudotime ordering of NSCs lineage development in adult SVZ datasets and scatter plots showing the expression of Gas1 and Mki67 along the pseudotime trajectory. Top, Silla and colleagues dataset (n = 14,660 cells). Bottom, Borrett and colleagues dataset (n = 3,045 cells). The original data underlying S5B, S5D, and S5F Fig can be found in S1 Data. (PDF) [file pbio.3003100.s005.pdf]

Figure S6

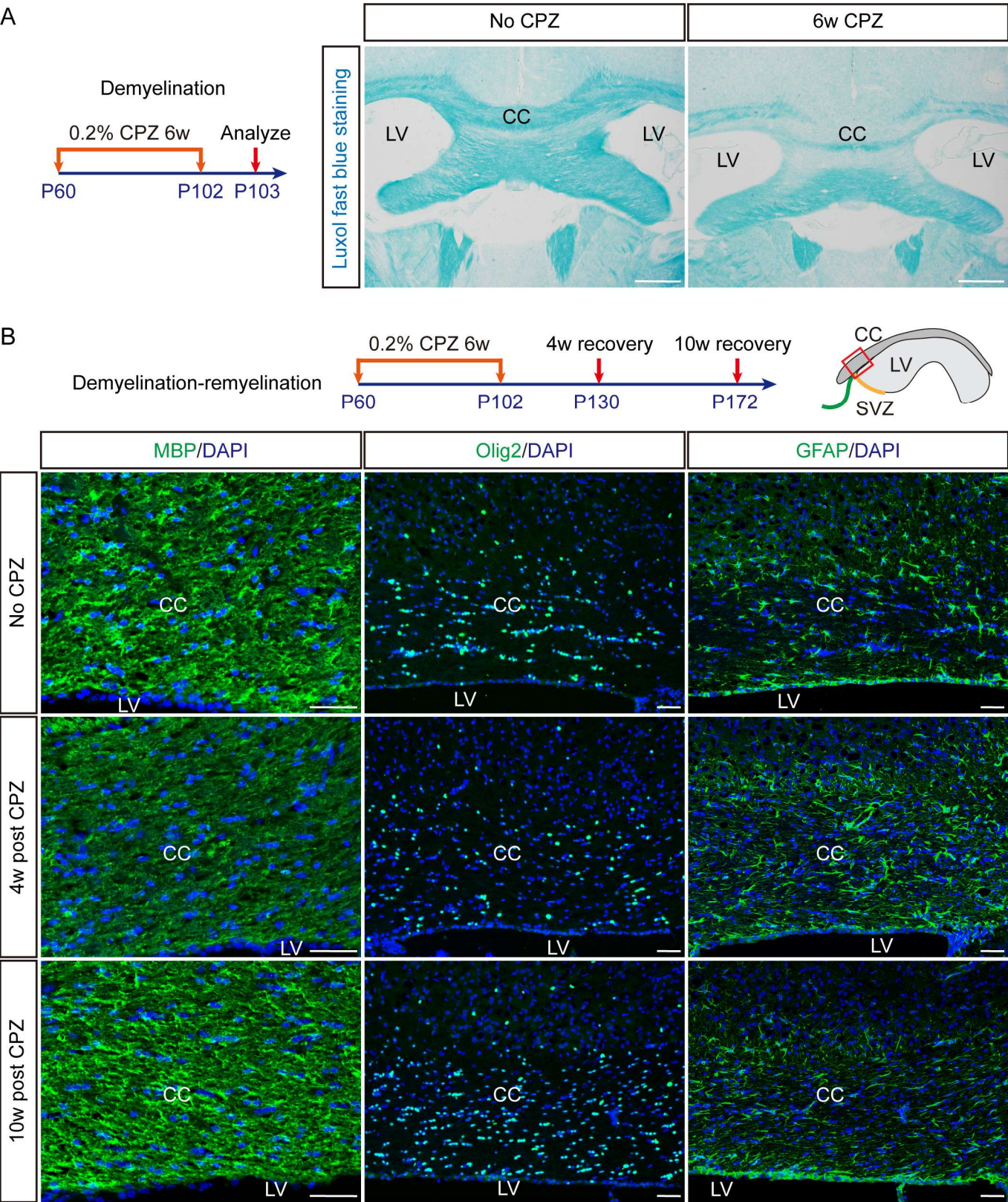

Supplement: S6 Fig — (A) Gas1 CreER/+ ; RT/+ mice at P60 were fed with normal (No CPZ) or 0.2% CPZ (6w CPZ) diet from P60 to P102 and analyzed at P103. Representative images of Luxol fast blue staining on corona brain sections from No CPZ and 6w CPZ groups are shown. Scale bar, 200 μm. (B) Gas1 CreER/+; RT/+ mice were fed with normal (No CPZ) or 0.2% CPZ in diet from P60 to P102, allowed to recover and analyzed at P130 (4 weeks post-CPZ) or P172 (10 weeks post-CPZ). Coronal brain sections from No CPZ, 4 and 10 weeks post-CPZ groups were IF stained for MBP, Olig2 or GFAP. Scale bars, 100 μm. LV, lateral ventricles; CC, corpus callosum; CPZ, cuprizone. (PDF) [file pbio.3003100.s006.pdf]

Uncropped gel for Fig. S3A

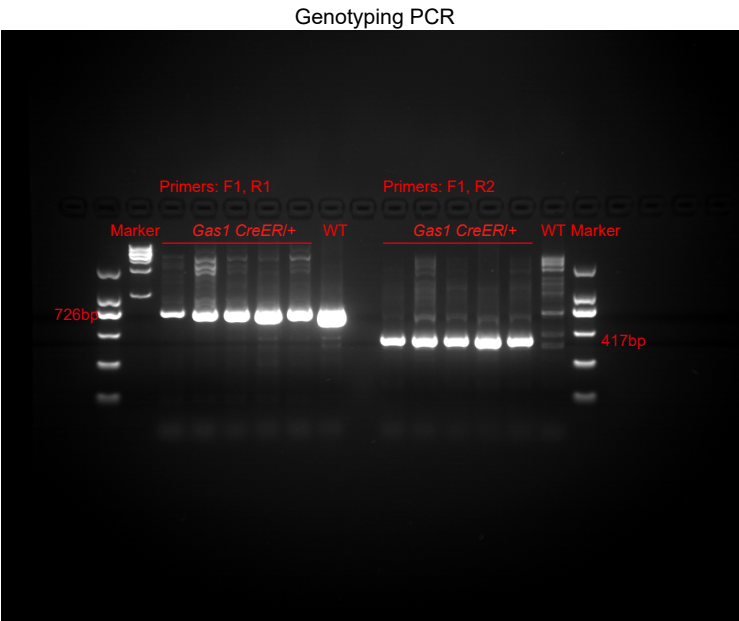

Uncropped gels for Fig. S3B

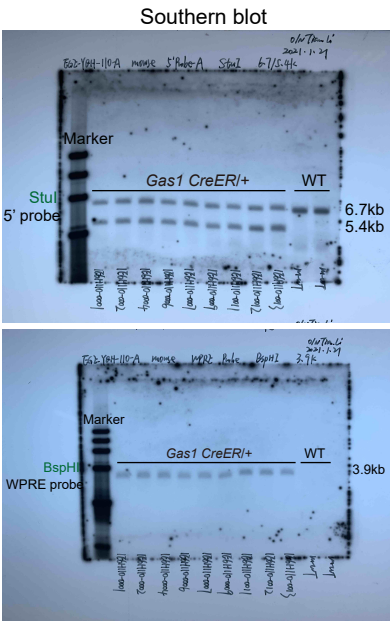

Supplement: S1 Raw Images — (PDF) [file pbio.3003100.s014.pdf]
