# Supplementary material for: Gas1-high quiescent neural stem cells are multipotent and produce oligodendrocytes during aging and after demyelinating injury
Source: PLoS Biol. 2025 Apr 3;23(4):e3003100. doi: 10.1371/journal.pbio.3003100 (PMC11990765; doi:10.1371/journal.pbio.3003100)
Supplement: Attachment — Submitted filename: Response to Reviewers.pdf [file pbio.3003100.s015.pdf]

Response to Reviewers' Comments (PBIOLOGY-D-24-02192R1)

We would like to thank both reviewers for their thorough and high-quality reviews. Their critiques have greatly helped to improve the manuscript. Following their constructive suggestions, we have substantially revised our manuscript and figures. Below, we provide a point-by-point rebuttal to the issues they raised. The revised text is highlighted in yellow in the manuscript.

Reviewer #1: "*This manuscript is focused on the role of growth-arrest-specific 1 (Gas1), a cell surface protein that can act as a modulator of Sonic hedgehog (SHH) signaling, within the prenatal and postnatal neural stem cell niche in the mouse. Mining prior sc-RNASeq datasets for discovery, the authors identify gas1 transcript as present in both eNSCs and adult NSCs, and argue that it is primarily present in cells with low ki67 transcript. Using a new CreER inducible reporter mouse model which is also hemizygous for Gas1, the authors conduct lineage tracing across multiple time intervals including early development and late adult life, and show that Gas1-CreER-labeled cells are present in prenatal and postnatal stages. The model advanced is that Gas1 regulates quiescent neural stem cells that first appear in the embryo and persist throughout adult life, and that alterations in Gas1 affect stem cell activation following demyelinating lesions or exogenous SHH administration. This work does* ***refine our understanding*** *of the potential role of Gas1 in the neural stem cell niche, and presents an* ***interesting contrast*** *to other members of the SHH pathway.*"

Response: Thank you for your summary and positive comments.

"*However, the data and model as presented are somewhat muddled - while the data that Gas1 is expressed by neural stem cells across the lifespan is convincing, there is not a strong, clear case that Gas1 is specifically involved in the quiescent population and not other cell types within the niche, and there is relatively limited data directly*

showing that its role is specific to dorsal SVZ. The experiments presented also do not clearly distinguish between potential roles in quiescence entry vs quiescence exit (while the text makes specific claims about one of these). Many of these concerns could be addressed by additional quantification, or alternative analyses, of the data included in the manuscript, as well as clearer depictions of the immunostaining data. The model for Gas1's functional role should also be clarified."

Response: Thank you for these insightful suggestions. We have performed additional experiments to address cell-type specificity issues and quiescence regulation by Gas1, along with other concerns as follows.

Major concerns:

1. "*Additional careful quantification of much of the imaging data, and inclusion of the quantification in the figures proper, would greatly aid readers' ability to interpret the data.*"

Response: We have carefully quantified most of the imaging data and included the quantifications in the figures proper.

2. "*In many cases, it's quite difficult to assess where in the SVZ or OB the representative images were obtained; it would be extremely helpful to indicate this more clearly with an overview low magnification image and/or cartoon. This is particularly important for this paper given the discussion of specific subdomains within the SVZ. Please include clear images of the ventral SVZ domain with reporter labeling and/or direct staining for Gas1.*"

Response: Thank you for these suggestions. We indicated the regions for each high-mag images with cartoons or low-mag overview. We also included the ventral SVZ domain with reporter labeling (Fig 3B) or direct antibody staining for Gas1 (Fig 1I. See also Response to Reviewer 2, Major Concern 1 and 2).

Fig. 3B and Fig. 1I

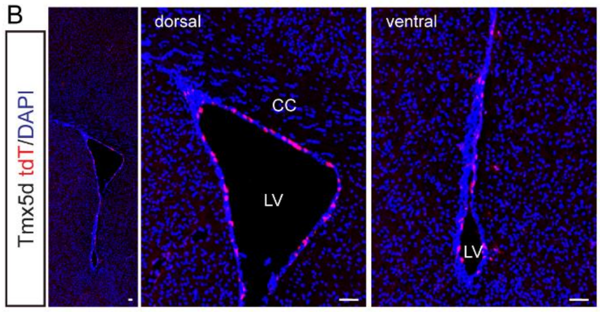

Text (Lines 219-224)

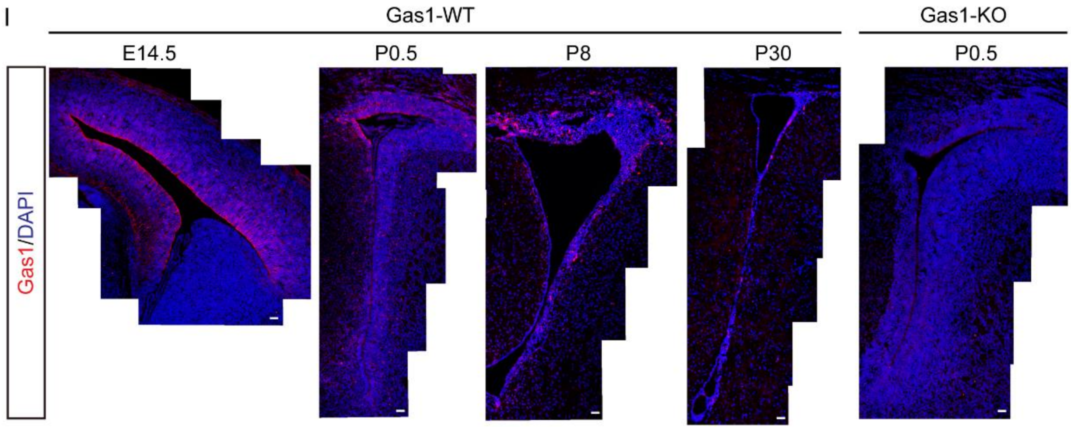

Text (Lines 147-157)

3. "*The proposed difference in G0/G1 versus S fraction upon overexpression of Gas1 is relatively small. It would strengthen confidence to include nucleoside analog incorporation (ie BrdU-PI biaxial flow cytometry data) as a more direct measurement of S phase entry. More broadly, there is the question of how to interpret these data - if overexpression of Gas1 leads to a decrease in S phase fraction, this seems more indicative of a role related to entry or maintenance of quiescence rather than a "gatekeeper to quiescence exit" as proposed by the authors. If Gas1 is essential for proper quiescence exit, a more appropriate experiment would potentially be to start with quiescence induction, then modulate Gas1, and determine whether successful re-entry to cell cycle occurs when quiescent cells are re-exposed to mitogens.*"

Response: Following your suggestions, we performed additional EdU/DAPI biaxial flow cytometry experiments. We first reanalyzed the overexpression experiments on

NSCs cultured from E14.5 VZ/SVZ. Overexpression of Gas1 leads to a decrease in S phase fraction similar to our previous results (Fig S5C-D). For knockout (KO) experiments, we chose postnatal NSCs cultured from P0.5 SVZ, which appear relatively more quiescent than E14.5 NSCs (with higher G0/1 ratios), and compared wildtype and Gas1-KO (Gas1 CreER/CreER) NSCs. We found that Gas1-KO NSCs exhibit increased S phase ratios (Fig S5E-F). We also found that few $Gas1^{+}$ NSCs are $Ki67^{+}$ in the adult SVZ (Fig 4A. ~5% versus ~20% in $Gas1^{-}$ cells), and they form smaller neurospheres compared to $Gas1^{-}$ NSCs when cultured in vitro (Fig 4B-C). With these new data, we agree with your assessment that the primary function of Gas1 in NSCs is quiescence maintenance, and have refined our interpretation accordingly.

Fig. S5C-F and Fig. 4A-C

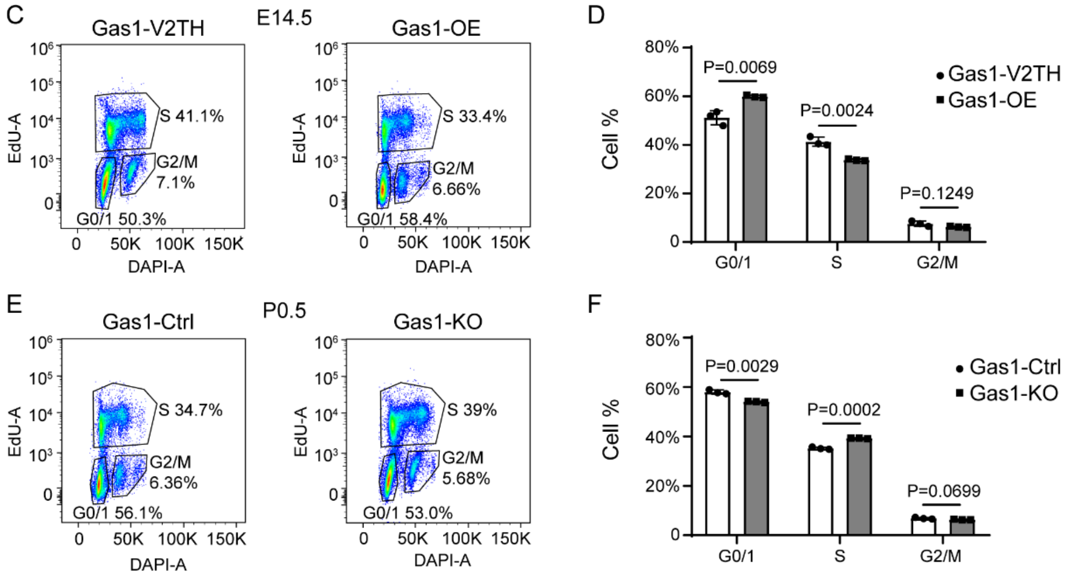

Text (Lines 249-254)

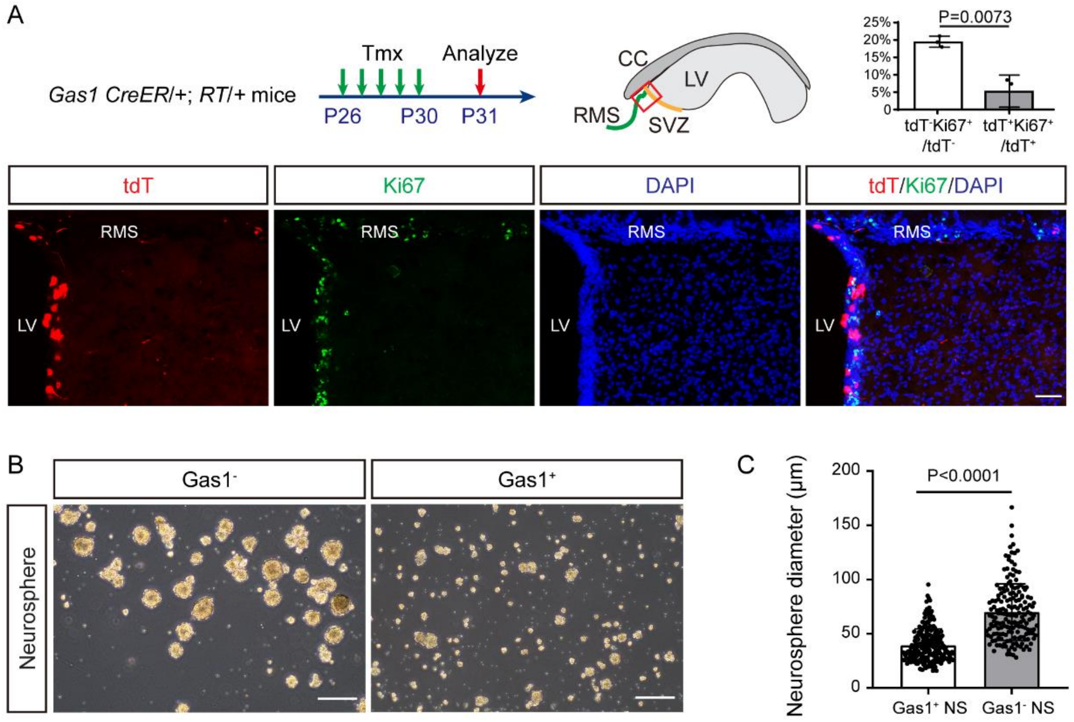

Text (Lines 254-256)

4. "*The lineage tracing and immunostaining data indicate that Gas1-expressing cells can produce oligodendrocytes and neurons (ie they are bipotent as suggested by the authors), but the production of astrocytes is not conclusively ruled out (ie it is hard to be certain that these cells are bipotent and not tripotent). This claim should potentially be tempered.*"

Response: We fully agree that it is difficult to rule out astrocytic differentiation and to be certain whether they are bipotent or tripotent. Accordingly, we changed "bipotent" to a more inclusive term "multipotent" throughout the manuscript.

5. "*For Gas1 magnetically-sorted cells - was any direct staining for Gas1 completed on the post-sort populations? Given the relatively modest shift in glial production in the positively and negatively sorted populations, this is important to confirm that the expected enrichment occurred.*"

Response: We performed additional experiments to validate the identity of

magnetically sorted cells, by staining sorted cells with Gas1 antibody for FACS analysis (Fig S5A). We found that around 90% of the sorted cells are indeed Gas1$^+$, confirming effective enrichment (Fig S5B).

Fig. S5A-B

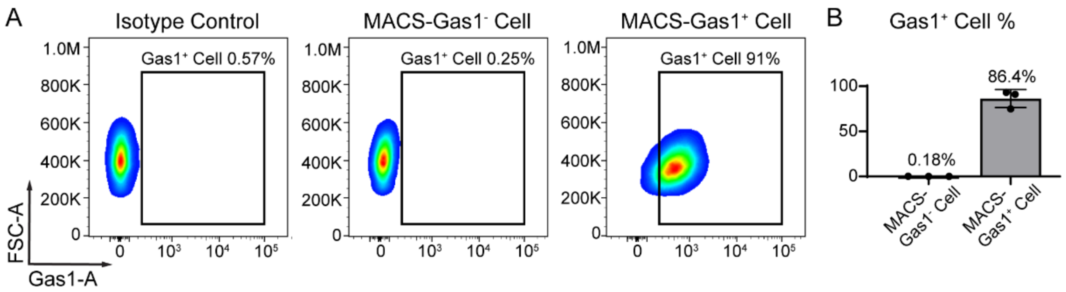

Text (Lines 242-243)

As for the differentiation experiments, we wouldn't call the shift in oligodendrocyte lineage production as "modest". In these standard serum-free spontaneous NSC differentiation assays, differentiation is induced by growth factor withdrawal (minus EGF/FGF) rather than adding differentiation factors, and the combined ratio of differentiated neuron and oligodendrocyte is often around 10%. Thus, the 3-fold increase of NG2$^+$ ratio from 1.5-2% to 5.5-6% is quite dramatic in these experimental settings.

Minor concerns:

1. "*Line 95 of text - suggest also citing Machold et al 2003 and Ihrie et al 2011*"

Response: We included both references in the revised manuscript. Text (Lines 95).

2. "*In several figures, when single-channel immunostaining is shown, it is blue on black - showing this in grayscale would greatly aid visualization*"

Response: Thank you for your suggestions. We changed all blue-on-black images into

grayscale.

3. "*Some data plots depict data using SEM to show the error - this is nearly always incorrect for experimental biology data. More appropriate is to show error bars as SD.*"

Response: We agree. We changed error bars in all data plots to SD, and revised the figure legends accordingly.

4. "*Figure S1 - please make the transcript labels on the plots more legible. Can you show Gas1 transcript levels on the plot from S1B?*"

Response: We split Fig. S1 into two supplemental figures (Fig S1 embryonic/postnatal datasets and Fig S2 adult datasets) to make each plot/label more legible. We also included Gas1 transcript levels on the plot from S1B (now Fig S2B), as well as Borrett et al. P61 data (now Fig S2A) to show consistent Gas1-expression patterns in the adult SVZ across datasets.

Fig. S1 and Fig. S2A-B

Text (Lines 110-131)

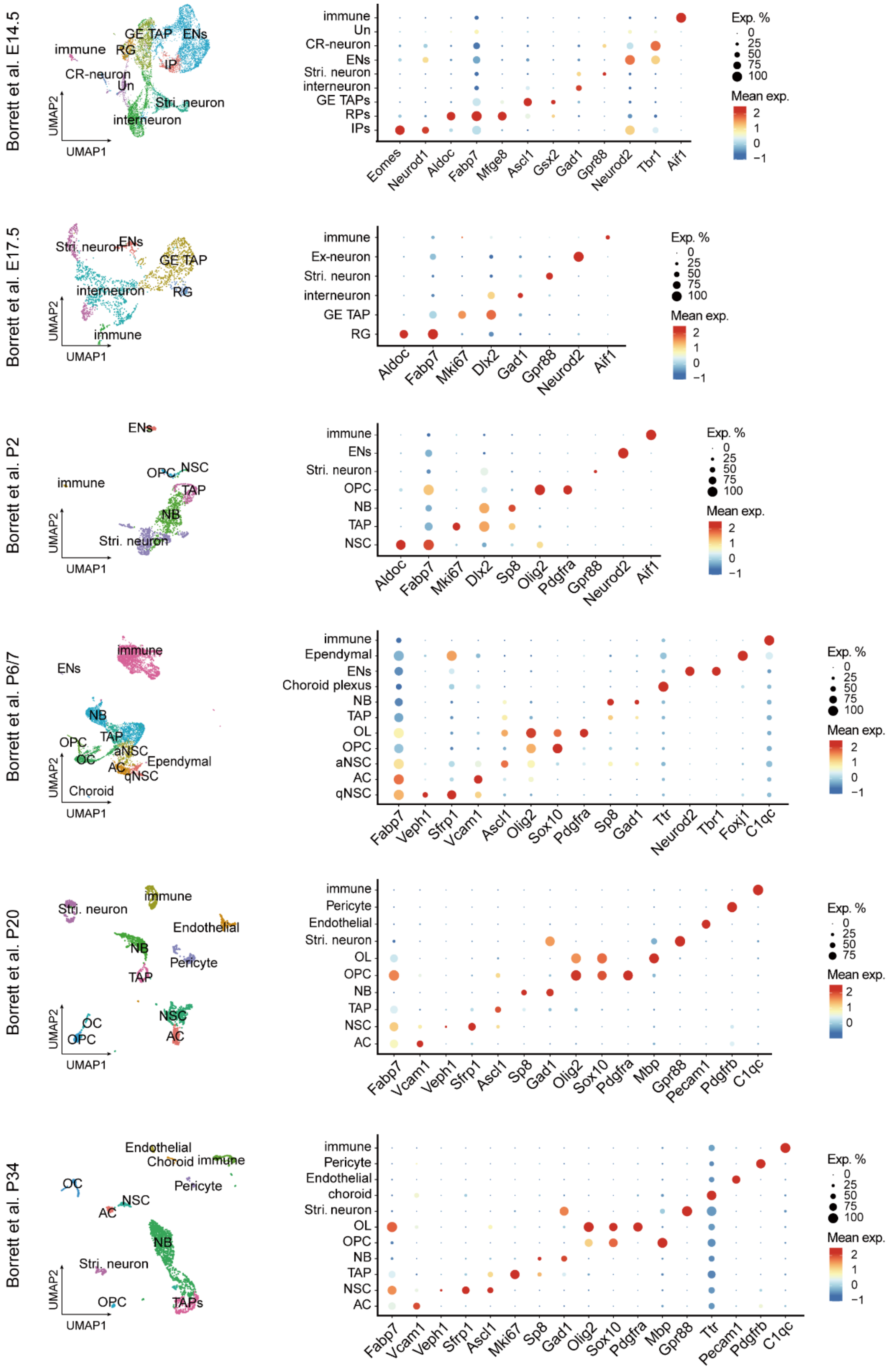

Borrett et al. E14.5
immune
RG
GE TAP
ENs
CR-neuron
IP
Un
Stri. neuron
interneuron
UMAP2
UMAP1
immune
Un
CR-neuron
ENs
Stri. neuron
interneuron
GE TAPs
RPs
IPs
Eomes
Neurod1
Aldoc
Fabp7
Mfge8
Ascl1
Gsx2
Gad1
Gpr88
Neurod2
Tbr1
Aif1
Exp. %
0
25
50
75
100
Mean exp.
2
1
0
−1
Borrett et al. E17.5
Stri. neuron
ENs
GE TAP
interneuron
RG
immune
UMAP2
UMAP1
immune
Ex-neuron
Stri. neuron
interneuron
GE TAP
RG
Aldoc
Fabp7
Mki67
Dlx2
Gad1
Gpr88
Neurod2
Aif1
Exp. %
0
25
50
75
100
Mean exp.
2
1
0
−1
Borrett et al. P2
ENs
OPC
NSC
immune
TAP
NB
Stri. neuron
UMAP2
UMAP1
immune
ENs
Stri. neuron
OPC
NB
TAP
NSC
Aldoc
Fabp7
Mki67
Dlx2
Sp8
Olig2
Pdgfra
Gpr88
Neurod2
Aif1
Exp. %
0
25
50
75
100
Mean exp.
2
1
0
−1
Borrett et al. P6/7
immune
ENs
NB
TAP
OPC
aNSC
OC
Ependymal
AC
qNSC
Choroid
UMAP2
UMAP1
immune
Ependymal
ENs
Choroid plexus
NB
TAP
OL
OPC
aNSC
AC
qNSC
Fabp7
Veph1
Sfrp1
Vcam1
Ascl1
Olig2
Sox10
Pdgfra
Sp8
Gad1
Ttr
Neurod2
Tbr1
Foxj1
C1qc
Exp. %
0
25
50
75
100
Mean exp.
2
1
0
−1
Borrett et al. P20
immune
Stri. neuron
Endothelial
NB
Pericyte
TAP
OC
OPC
NSC
AC
UMAP2
UMAP1
immune
Pericyte
Endothelial
Stri. neuron
OL
OPC
NB
TAP
NSC
AC
Fabp7
Vcam1
Veph1
Sfrp1
Ascl1
Sp8
Gad1
Olig2
Sox10
Pdgfra
Mbp
Gpr88
Pecam1
Pdgfrb
C1qc
Exp. %
0
25
50
75
Mean exp.
2
1
0
Borrett et al. P34
Endothelial
Choroid
immune
OC
Pericyte
NSC
AC
NB
Stri. neuron
OPC
TAPs
UMAP2
UMAP1
immune
Pericyte
Endothelial
choroid
Stri. neuron
OL
OPC
NB
TAP
NSC
AC
Fabp7
Vcam1
Veph1
Sfrp1
Ascl1
Mki67
Sp8
Gad1
Olig2
Sox10
Pdgfra
Mbp
Gpr88
Ttr
Pecam1
Pdgfrb
C1qc
Exp. %
0
25
50
75
100
Mean exp.
2
1
0
−1

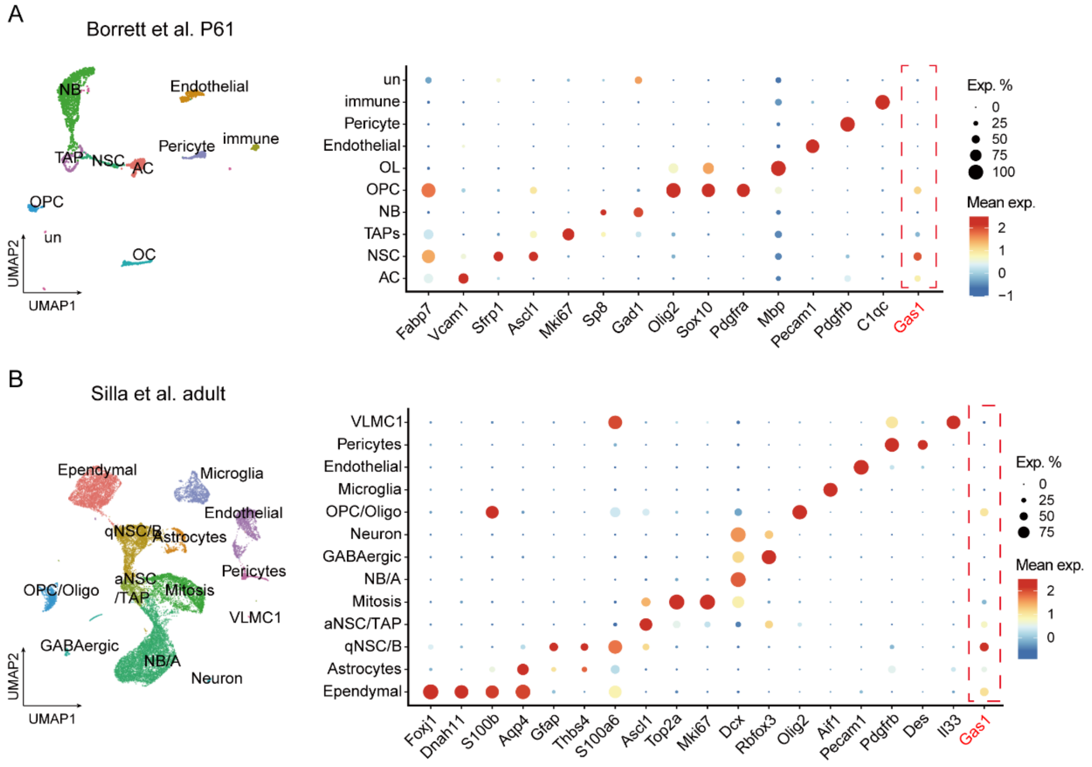

5. “*Figure 2F and 2G - how are the boundaries of the VZ/SVZ defined? This is essential to understanding the proposed difference in the quantification.*”

Response: At P0.5, postnatal NSCs/NPCs reside in the VZ/SVZ area with high cellular density distinct from the surrounding brain regions (Tramontin et al., *Cerebral Cortex*, 2003). Thus, in Figure 2F and 2G we delineated the boundaries of VZ/SVZ mainly based on the density of DAPI-stained nuclei.

Reviewer #2: "*This study characterizes a subset of quiescent dorsal neural stem cells (NSCs) in the subventricular zone (SVZ) identified by their expression of Gas1, a co-receptor of Patched. By mining published scRNAseq data bases, they identified Gas1 as a marker of NSCs and some ependymal cells with limited overlap with more differentiated lineages. They then generated a Gas1-CreER driver line that allowed them to report Gas1+ cells, carry out scRNAseq, analyze neurospheres, and generate knockout mice and fate map after demyelination. They report that Gas1+ cells are quiescent (q) NSCs, with enhanced gliogenic potential, that are a significant source of oligodendrocyte and following cuprizone-mediated demyelination. Overall, while* ***potentially very interesting****, I have several concerns that limit my enthusiasm for the study in its current form.*"

Response: Thank you for your summary and thoughtful critiques.

Major concerns:

1. "*Chief among these is whether the fate mapped Gas1+ cells exclusively derive from the SVZ as implied or may also include parenchymal cells that confound their analysis of remyelination. Their data shows their Cre driver reports cells in the SVZ and ependyma, with many hi mag images of the (dorsal) SVZ. However, two separate data bases: https://brainrnaseq.org and http://dropviz.org indicate there is robust expression of Gas1 in astrocytes and OPCs. While astrocytes are unlikely to confound their analysis of remyelination, OPCs certainly would. I am having difficulty reconciling the notion that Gas1+ cells are only in the SVZ with these other data bases. A young adult mice treated with TMX x5 (as in Figure S2C) that then shows a much wider field of view and includes staining for GFAP and NG2 and shows the CC and cortical layers, and the full ventral and dorsal extent of the SVZ would be helpful. If these cells are truly only in the SVZ, how do they reconcile their fate mapping with these data bases? RNAscope to address expression outside of the SVZ might also aid in addressing this discrepancy as well.*"

Response: We appreciate your independent analysis of Gas1 expression and valuable suggestions. We did not observe tdT$^+$ cells outside the SVZ from mice treated with 5x TMX (Fig 3B). Gas1 antibody immunostaining also confirms that Gas1 protein is barely expressed outside the SVZ, in particular the corpus callosum where a large number of astrocytes and OPCs reside (Fig 1I).

Fig. 3B and Fig. 1I

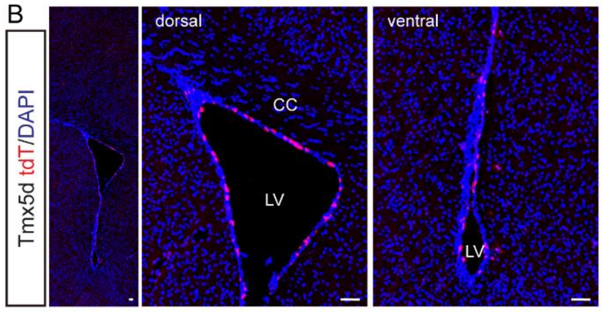

Text (Lines 219-224)

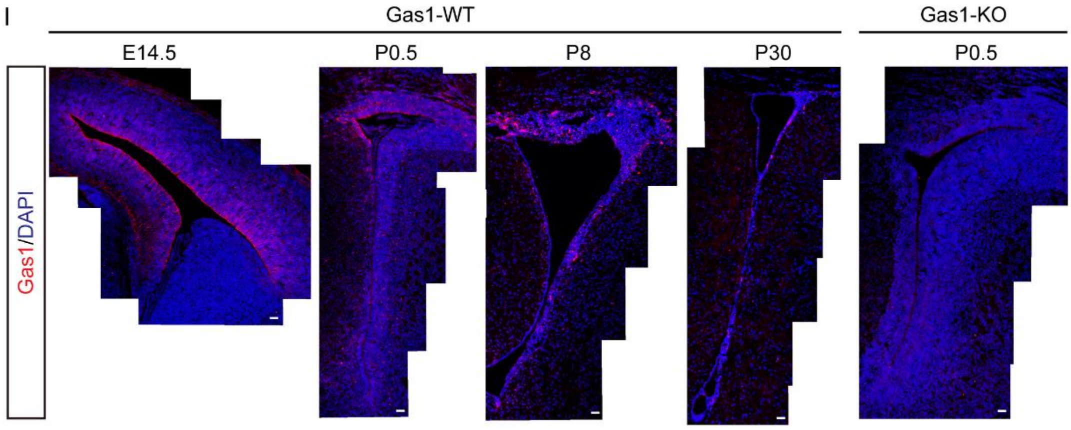

Text (Lines 147-157)

One possible explanation to reconcile these observations is that similar to certain Cre lines, our Gas1-Cre^{ER} system only label cells with high Gas1 expression (Gas1^{high}) but rarely Gas1^{low} cells. We first checked the two databases you mentioned (Rebuttal figure 1). As you said, Gas1 is expressed in both astrocytes and OPCs, higher than other cell types in the cortex. However, these are scaled/normalized expression data and neither of them include SVZ NSCs, thus cannot be used to compare Gas1

expression in astrocytes/OPCs versus NSCs (Rebuttal figure 1). In two independent single-cell RNA-seq datasets that include SVZ NSCs (Fig S2A-B), we found while Gas1 is expressed in OPCs and astrocytes, its expression level is highest in NSCs, supporting the notion that our Gas1-Cre^{ER} system only label Gas1^{high} cells.

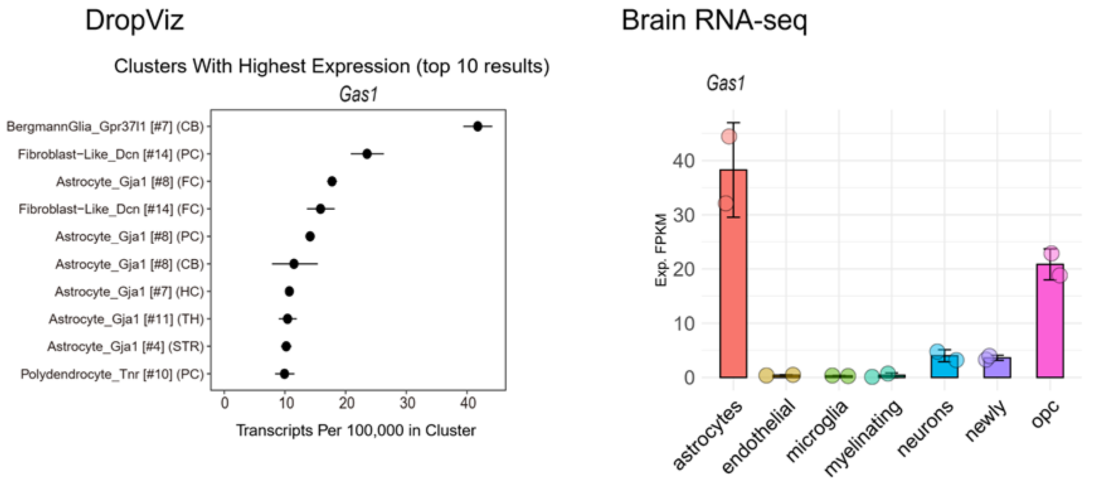

Rebuttal figure 1. Expression of Gas1 in public datasets, DropViz (Left) and Brain RNA-seq (Right).

Fig. S2A-B

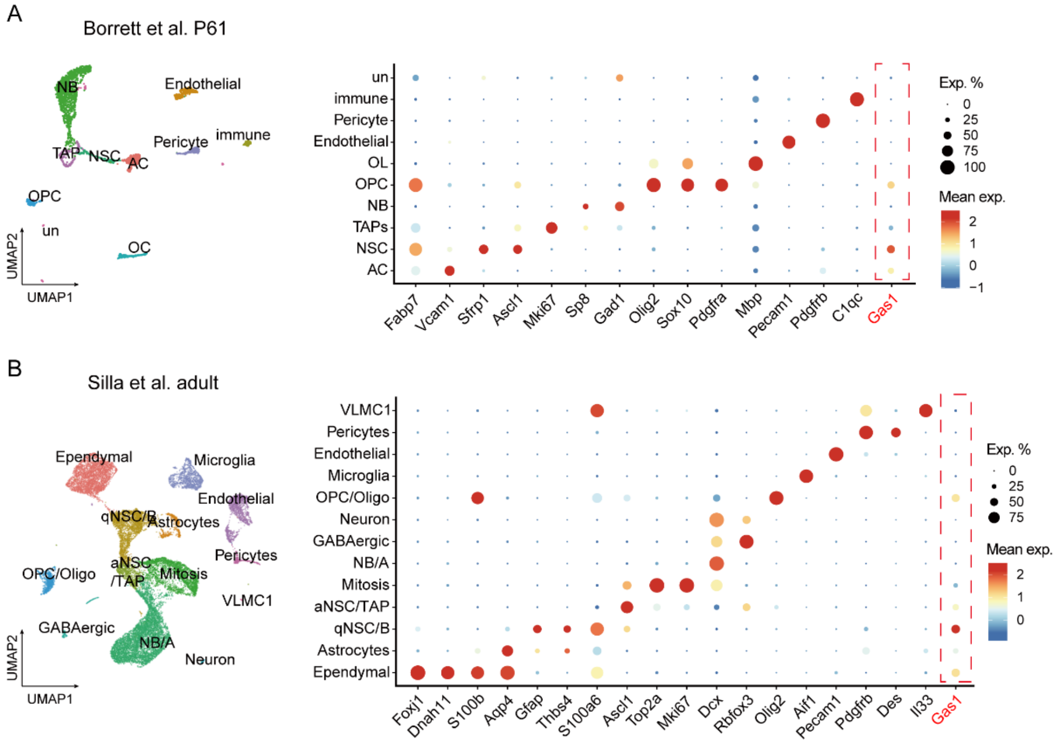

Text (Lines 112-131)

2. “*A related issue - that Gas1+ cells are principally localized dorsally - could be stronger. Gas1 works cooperatively with Patched to promote SHH signaling and in other settings is co- expressed with Gli1 so the notion that these mark divergent NSC populations i.e., dorsal vs ventral SVZ, respectively is perhaps unexpected but interesting. (The limited Gli1 expression by Gas+ cells in Fig. 5E is not surprising given they analyzed only the dorsal/lateral SVZ.) In situs/RNAscope would be useful to show these are spatially distinct populations - in particular that Gas1 is largely dorsal. Alternatively, short term fate mapping with the Gas1 Cre driver in adults and imaging the entire SVZ as above could strengthen this key point. If the Gas1 antibodies are reliable, this could be done by immunostaining and address the question of whether other cells (astrocytes, OPCs) are also Gas1+. Demonstrating the Gas1 antibodies fails to stain the SVZ in nullizygous mice (Gas1 cre/cre) would be useful in this regard. The authors show the Gas1-high cells proliferate with Shh injection, do they also become Gli1+ after injection?*”

Response: Thank you for your insightful suggestions. We further validated the cell type and regional specificity using Gas1 antibody staining and short-term fate mapping. In the Gas1 staining experiment, we first confirmed the specificity of the antibody, which fails stain the Gas1-KO SVZ (Gas1 CreER/CreER) at P0.5 but exhibits robust staining in the wildtype SVZ. At P30, we imaged the entire SVZ and found that Gas1 antibody-stained cells are mostly in the dorsal SVZ, but not in the surrounding corpus callosum where astrocytes and OPCs/OCs reside (Fig 1I). These observations are consistent with our previous conclusion that Gas1^{high} cells are largely NSCs in the dorsal SVZ.

Fig. 1I.

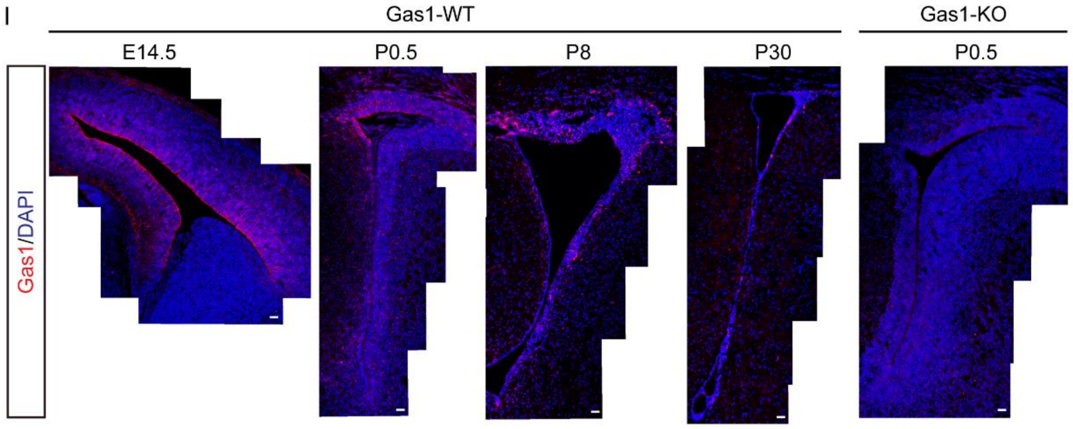

Text (Lines 147-157)

In the short-term fate mapping experiment, we imaged the entire SVZ to show the overall the distribution of $Gas1^{high}$ tdT^{+} cells, which are also largely dorsal (Fig 3B). Similar to antibody staining, we did not observe tdT^{+} cells in the corpus callosum (Fig 3B). Notably, our reporter system appears to label quite a few ependymal cells lining up the ventricular wall as we initially reported, which is different from the antibody staining pattern (Fig 1I). This could be explained by the differential regulation of Gas1 at the protein versus mRNA levels. For the few tdT^{+} cells in the ventral SVZ, we double-checked their identity and found that they are mostly (~90%) $Foxj1^{+}$ ependymal cells (Fig 3C).

Fig. 3B-C

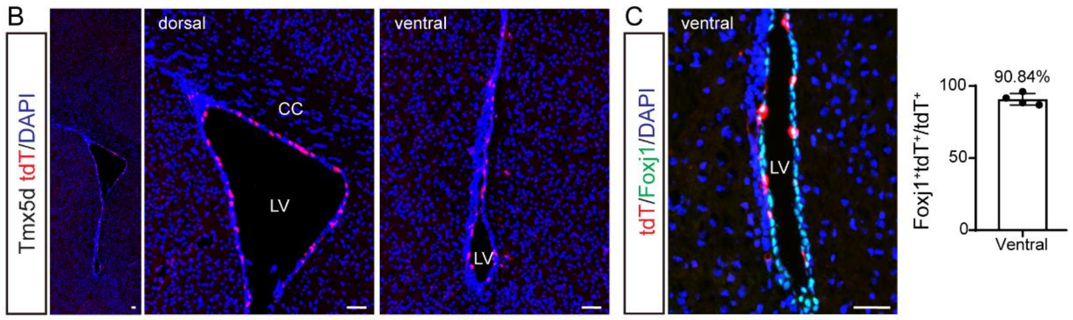

Text (Lines 219-224)

To address your question whether Gas1^{high} cells become Gli1^{+} after SHH treatment, we magnetically sorted Gas1^{high} and Gas1^{low} cells from pooled SVZ cells at P30, treated them with SHH, and performed qPCR to compare their Gli1 expression levels (Fig 4F). Consistent with the scRNA-seq data, Gli1 expression is very low in Gas1^{high} cells, but increased to control levels after SHH treatment.

Fig. 4F

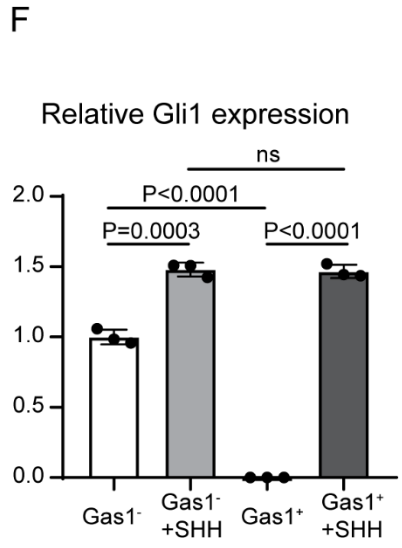

Text (Lines 256-259)

In summary, Gas1^{high} cells are mostly NSCs in the dorsal SVZ not in ACs or OPCs in the corpus callosum, evidenced by both antibody staining and fate mapping experiments, although our fate mapping system does label a subset of ependymal cells, these cells are not generally considered as gliogenic thus do not complicate our main conclusions.

3. "*The authors suggest Gas1+ cells are quiescent in part based on finding that expression of Gas1 in (Gas1-) NSCs inhibits a transition from G0/G1 to the S phase. Consistent, Fig 5C classification suggests Gas1+ cells have somewhat lower Nestin levels. However, immunostaining of the SVZ in Aged brains indicates many of these cells are Nestin+. A more complete characterization of their scRNA seq*

defined populations using other markers i.e., EGFR for aNSCs and Lrig1 & PDGFRbeta as markers of qNSCs would be useful to assess this classification."

Response: The relative quiescence of Gas1^{+} cells is confirmed by additional experiments (See also Response to Reviewer 1, Major Concern 3). The original Fig.5C shows scaled Nes expression compared to other cell types, which does not mean it is not expressed in the Gas1^{+} population. In the revised Fig. 6, we performed a more complete characterization using suggested markers including EGFR, Lrig1 and PDGFRbeta, redefined cell populations based on these markers, and reanalyzed the gene expression and gene ontology based on new cell identities. The results are largely consistent with our initial report.

Fig. 6

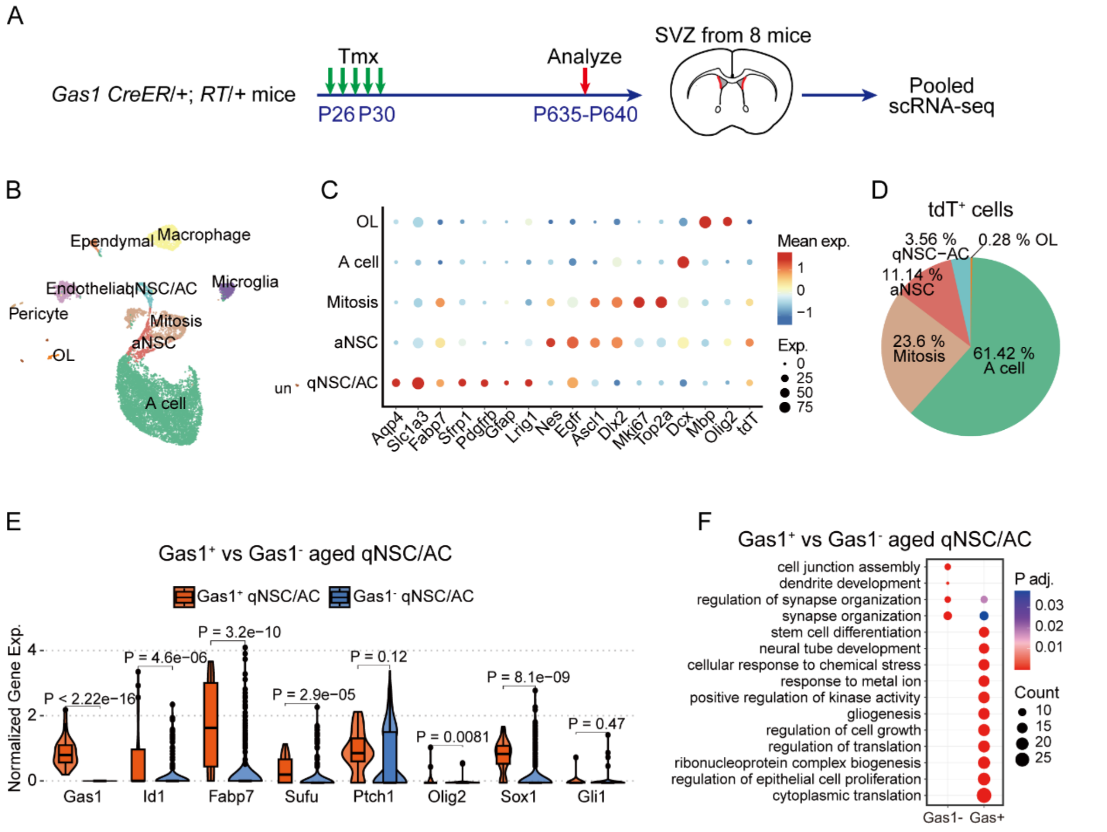

Text (Lines 273-286)

4. "*It is unclear to this reader that the pseudotime for Gas1 shown in Fig. 1 actually indicates it is downregulated. RNAscope or immunostaining for Gas1 at different time points would be useful to corroborate the pseudotime analysis. The limited cell numbers used suggest the analysis is underpowered. As a minor point, it is unclear why the P6/7 color in key is listed between P34 and P61 rather than in the correct temporal order. It is also the only data cohort corresponding to qNSC so should probably not be included in any case.*"

Response: We performed immunostaining for Gas1 at different time points (E14.5, P0.5, P8 and P30) to corroborate the pseudotime analysis, which exhibits consistent downregulation of Gas1 in the VZ or SVZ regions (Fig 1I). We also corrected the order of the P6/7 color in key to reflect the correct temporal order (Fig 1A-C). We chose to keep this data cohort since it is consistent with our P8 staining pattern (Fig 1I).

Fig. 1A-C, I

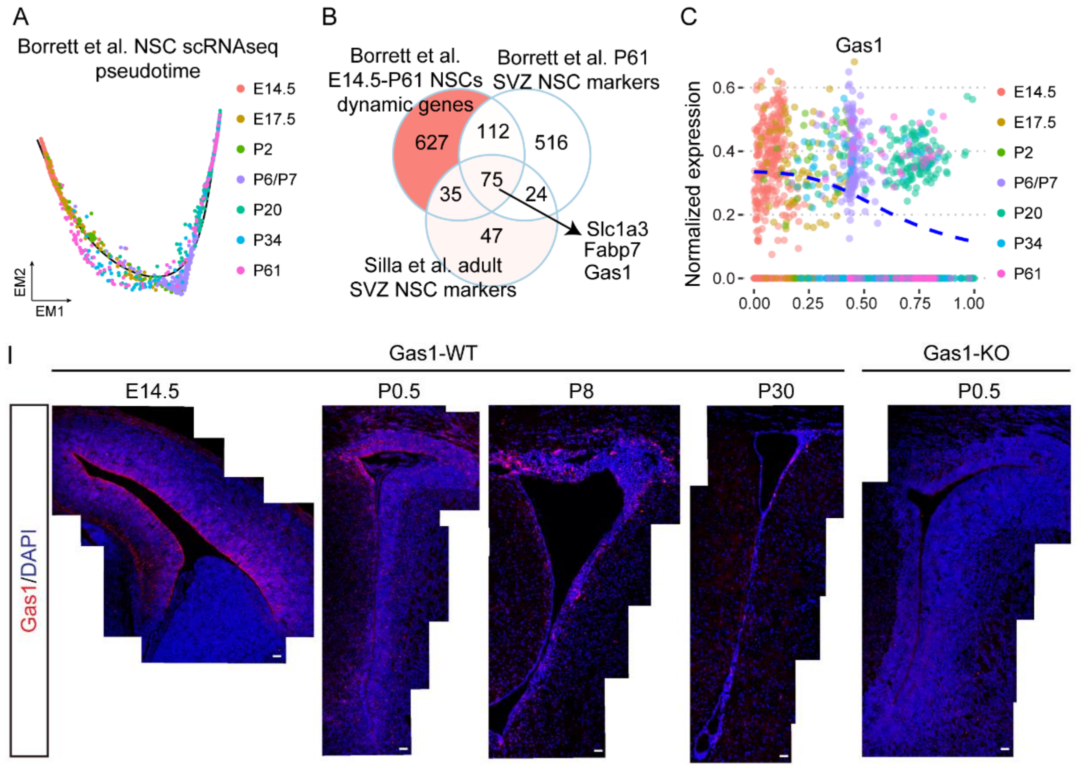

Text (Lines 147-157)

5. “*The increase of TdT labeled cells in the CC post-cuprizone shown in Fig. 6 is very striking. This key point would be greatly strengthened with better images - the ones shown are suboptimal and out of focus. Given the extensive CNPase staining in the field, the precise overlap of these markers is unclear from their images. It is unclear from the legend and methods how these images were generated but confocal analysis would be helpful to corroborate the overlap of markers and potentially assess if TdT is present in myelin sheaths co-stained for MBP.*”

Response: Thank you for your suggestions. Indeed, these images were captured by the Olympus BX51 regular fluorescent microscope. To corroborate the overlap of oligodendrocyte markers, we performed additional immunostaining of CNPase and MBP, and imaged the slides using a confocal microscope (Fig 7B-C). A large number of tdT^+ cells colocalize with CNPase, and tdT is present in MBP^+ myelin sheaths, consistent with the notion that $Gas1^{high}$ cells generate mature oligodendrocytes.

Fig. 7B-C

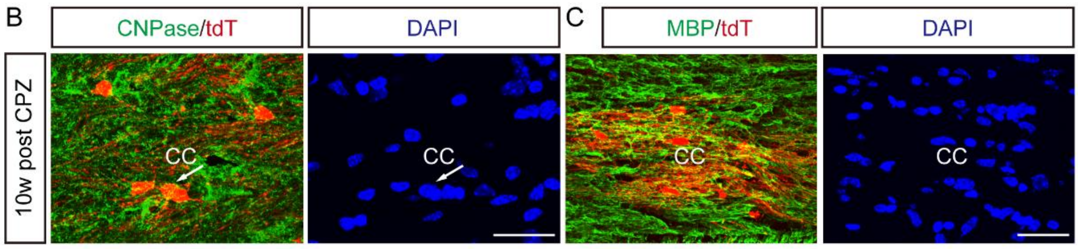

Text (Lines 304-306)
