# Supplementary material for: Gas1-high quiescent neural stem cells are multipotent and produce oligodendrocytes during aging and after demyelinating injury
Source: PLoS Biol. 2025 Apr 3;23(4):e3003100. doi: 10.1371/journal.pbio.3003100 (PMC11990765; doi:10.1371/journal.pbio.3003100)
Supplement: Attachment — Submitted filename: Response to Reviewers.docx [file pbio.3003100.s017.docx]

Response to Reviewers' Comments (PBIOLOGY-D-24-02192R2)

We would like to again thank both reviewers for their suggestions. Below, we provide a point-by-point rebuttal to remaining issues. The revised text is highlighted in yellow in the manuscript.

Reviewer #1: *The authors have* ***largely addressed*** *my points through rewording and additional experiments and quantification.*

Response: Thank you for your comments.

**Minor points:**

1. *Lines 206-207: Sox2 on its own does not distinguish quiescent NSCs - this should be phrased differently in the text.*

Response: We agree. We rephased the sentence to remove “qNSC” and focus on describing the results (Line 205-207).

2. *Line 1052 (figure legend): "corpus callosum" not "callous corpus"*

Response: Thank you and we corrected the typo in the figure legends.

3. *No reference to some of the panels from Figure 5 in the main text?*

Response: We made sure both panels from Figure 5 are referenced in the main text (Line 265-271).

Reviewer #2: *The authors have been* ***responsive*** *to the concerns raised by the reviewers. In particular, they have provided additional data that* ***strongly supports*** *that Gas1(high)+ cells are quiescent neural stem cells in the dorsal lateral SVZ with enhanced gliogenic potential and a significant source of demyelinating oligodendrocytes following cuprizone-mediated demyelination.*

Response: Thank you for your comments.

*My one remaining concern is the incomplete characterization of fate-mapped cells during remyelination shown in Fig. 7. There is an ~ 5x increase in Olig2+/TdT+ cells from 4 weeks to 10 weeks in the CC. The magnitude of this increase is surprising given that substantial re-myelination has likely already occurred by 4 weeks. It would be useful to know whether the total number of TdT+ cells in the CC at 4 weeks and 10 weeks show a comparable increase i.e., is this an increase in TdT+ cell numbers, upregulation of Olig2, or both? A full breakdown of the percentages of TdT+ cells that are OPCs, oligodendrocytes, astrocytes or cannot be classified at 4 and 10 weeks would provide additional context to this result and whether it is due to ongoing recruitment of stem cells or their ongoing differentiation.*

Response: We did have the quantification for the total tdT^+^ cells and the percentage of Olig2^+^tdT^+^ among total tdT+ cells at each timepoint, and have revised Fig. 7D and main text to include these data. Indeed, there is an increase in total tdT^+^ cell number as well as the percentage of Olig2^+^tdT^+^ cells (Line 304-307).

While we agree a full breakdown of tdT^+^ cells into OPCs, oligodendrocytes and astrocytes could provide additional context to these results, some of our sections (the 4-week group) are over 1 year old and suboptimal for immunostaining of additional lineage markers, and it is time consuming to repeat this experiment (6-week CPZ+4-week recovery). We believe the revised Fig. 7D is sufficient to address the reviewer’s main concern and support that there is continuous recruitment (increased tdT^+^ cells) and differentiation (increased Olig2^+^tdT^+^%) of stem cells.
