# Supplementary material for: Gas1-high quiescent neural stem cells are multipotent and produce oligodendrocytes during aging and after demyelinating injury
Source: PLoS Biol. 2025 Apr 3;23(4):e3003100. doi: 10.1371/journal.pbio.3003100 (PMC11990765; doi:10.1371/journal.pbio.3003100)
Supplement: Peer review history

# Author comment: Author response to Decision Letter 0

**DOI:** 10.1371/journal.pbio.3003100.r001

12 Jan 2025

Attachment Submitted filename: Response to Reviewers.pdf

# Editor report: Decision Letter 0

**DOI:** 10.1371/journal.pbio.3003100.r002

**Authors:** Lucas Smith

15 Jan 2025

Dear Dr Wang, 

Thank you for submitting your revised manuscript entitled "Gas1 integrates quiescence and multipotency in a neural stem cell subpopulation during aging and myelin repair" for consideration as a Research Article by PLOS Biology.

Your manuscript has now been evaluated by the PLOS Biology editorial staff and I am writing to let you know that we would like to send your submission back to the original reviewers. 

However, before we can send your manuscript to reviewers, we need you to complete your submission by providing the metadata that is required for full assessment. To this end, please login to Editorial Manager where you will find the paper in the 'Submissions Needing Revisions' folder on your homepage. Please click 'Revise Submission' from the Action Links and complete all additional questions in the submission questionnaire.

Once your full submission is complete, your paper will undergo a series of checks in preparation for peer review. After your manuscript has passed the checks it will be sent out for review. To provide the metadata for your submission, please Login to Editorial Manager (https://www.editorialmanager.com/pbiology) within two working days, i.e. by Jan 17 2025 11:59PM.

During the process of completing your manuscript submission, you will be invited to opt-in to posting your pre-review manuscript as a bioRxiv preprint. Visit http://journals.plos.org/plosbiology/s/preprints for full details. If you consent to posting your current manuscript as a preprint, please upload a single Preprint PDF. 

Feel free to email us at plosbiology@plos.org if you have any queries relating to your submission.

Kind regards,

Luke

Lucas Smith, Ph.D.

Senior Editor

PLOS Biology

lsmith@plos.org

# Author comment: Author response to Decision Letter 0

**DOI:** 10.1371/journal.pbio.3003100.r003

15 Jan 2025

Attachment Submitted filename: Response_to_Reviewers_auresp_1.pdf

# Peer review: Decision Letter 1

**DOI:** 10.1371/journal.pbio.3003100.r004

**Authors:** Lucas Smith

26 Feb 2025

Dear Dr Wang,

Thank you for your patience while we considered your revised manuscript "Gas1 integrates quiescence and multipotency in a neural stem cell subpopulation during aging and myelin repair" for consideration as a Research Article at PLOS Biology. Your revised study has now been evaluated by the PLOS Biology editors, the Academic Editor and the original reviewers who are laregly satisfied by the revision, but identify a few last issues that we think should be addressed. 

In light of the reviews, which you will find at the end of this email, we are pleased to offer you the opportunity to address the remaining point from the reviewers in a revision that we anticipate should not take you very long. We will then assess your revised manuscript and your response to the reviewers' comments with our Academic Editor aiming to avoid further rounds of peer-review, although we might need to consult with the reviewers, depending on the nature of the revisions.

**IMPORTANT: As you address the last reviewer comments, please also address the following editorial requests: 

1) TITLE: After some discussion within the team, we would like to propose a change to the title. If you agree, we suggest you change the title to: 

Quiescent neural stem cells that express high levels of Gas1 are multipotent and produce oligodendrocytes during aging and after demyelinating injury

or

Gas1-high quiescent neural stem cells are multipotent and produce oligodendrocytes during aging and after demyelinating injury

2) FINANCIAL DISCLOSURES: Please update your financial disclosures statement, in our online system, to indicate whether the sponsors or funders of your study played any role in the study design, data collection and analysis, decision to publish, or preparation of the manuscript.

3) ETHICS STATEMENT: Please update your ethics statement in the method section to include the specific national or international regulations/guidelines to which your animal care and use protocol adhered. Please note that institutional or accreditation organization guidelines (such as AAALAC) do not meet this requirement.

4) DATA: Thank you for providing the source data for your figures as a supplemental file. Please add a sentence to each figure legend pointing readers to this file. For example, you can add the sentence "the data underling this file can be found in __"

5) DATA: Thank you for depositing your data to NCBI Bioproject w/ the accession code PRJNA1107991. For some reason I could not access this dataset. Is it currently private? If so, please provide me with a reviewer token so I can check that it meets our reporting requirements (sorry if I missed this somewhere!). The dataset will need to be made public before publication. 

6) DATA: For figures containing flow cytometry data data, we ask that you provide FCS files and a picture showing the successive plots and gates that were applied to the FCS files to generate the figure. As these files are usually quite big, you can deposit them in the Flow Repository (http://flowrepository.org/) and please make sure they are publicly available. 

I have heard that flow repository has been giving people trouble lately - and so if you encounter issues with depositing this data, you can also put it on another publicly available repository. 

7) BLOT AND GEL REPORTING REQUIREMENTS: Please note that we require the original, uncropped and minimally adjusted images supporting all blot and gel results reported in an article's figures or Supporting Information files. Please prepare and upload them as a supplemental file according to our guidelines, which can be found here: https://journals.plos.org/plosbiology/s/figures#loc-blot-and-gel-reporting-requirements

We expect to receive your revised manuscript within 1 month. Please email us (plosbiology@plos.org) if you have any questions or concerns, or would like to request an extension. 

At this stage, your manuscript remains formally under active consideration at our journal; please notify us by email if you do not intend to submit a revision so that we withdraw the manuscript.

**IMPORTANT - SUBMITTING YOUR REVISION**

Your revisions should address the specific points made by each reviewer. Please submit the following files along with your revised manuscript:

1. A 'Response to Reviewers' file - this should detail your responses to the editorial requests, present a point-by-point response to all of the reviewers' comments, and indicate the changes made to the manuscript. 

*NOTE: In your point-by-point response to the reviewers, please provide the full context of each review. Do not selectively quote paragraphs or sentences to reply to. The entire set of reviewer comments should be present in full and each specific point should be responded to individually.

You should also cite any additional relevant literature that has been published since the original submission and mention any additional citations in your response. 

2. In addition to a clean copy of the manuscript, please also upload a 'track-changes' version of your manuscript that specifies the edits made. This should be uploaded as a "Revised Article with Changes Highlighted " file type. 

*Resubmission Checklist*

When you are ready to resubmit your revised manuscript, please refer to this resubmission checklist: https://plos.io/Biology_Checklist

To submit a revised version of your manuscript, please go to https://www.editorialmanager.com/pbiology/ and log in as an Author. Click the link labelled 'Submissions Needing Revision' where you will find your submission record. 

Please make sure to read the following important policies and guidelines while preparing your revision:

*Published Peer Review*

Please note while forming your response, if your article is accepted, you may have the opportunity to make the peer review history publicly available. The record will include editor decision letters (with reviews) and your responses to reviewer comments. If eligible, we will contact you to opt in or out. Please see here for more details:

https://blogs.plos.org/plos/2019/05/plos-journals-now-open-for-published-peer-review/

*PLOS Data Policy*

Please note that as a condition of publication PLOS' data policy (http://journals.plos.org/plosbiology/s/data-availability) requires that you make available all data used to draw the conclusions arrived at in your manuscript. If you have not already done so, you must include any data used in your manuscript either in appropriate repositories, within the body of the manuscript, or as supporting information (N.B. this includes any numerical values that were used to generate graphs, histograms etc.). For an example see here: http://www.plosbiology.org/article/info%3Adoi%2F10.1371%2Fjournal.pbio.1001908#s5

*Blot and Gel Data Policy*

We require the original, uncropped and minimally adjusted images supporting all blot and gel results reported in an article's figures or Supporting Information files. We will require these files before a manuscript can be accepted so please prepare them now, if you have not already uploaded them. Please carefully read our guidelines for how to prepare and upload this data: https://journals.plos.org/plosbiology/s/figures#loc-blot-and-gel-reporting-requirements

*Protocols deposition*

To enhance the reproducibility of your results, we recommend that if applicable you deposit your laboratory protocols in protocols.io, where a protocol can be assigned its own identifier (DOI) such that it can be cited independently in the future. Additionally, PLOS ONE offers an option for publishing peer-reviewed Lab Protocol articles, which describe protocols hosted on protocols.io. Read more information on sharing protocols at https://plos.org/protocols?utm_medium=editorial-email&utm_source=authorletters&utm_campaign=protocols

Thank you again for your submission to our journal. We hope that our editorial process has been constructive thus far, and we welcome your feedback at any time. Please don't hesitate to contact us if you have any questions or comments.

Sincerely,

Luke

Lucas Smith, Ph.D.

Senior Editor

PLOS Biology

lsmith@plos.org

----------------------------------------------------------------

REVIEWS:

Reviewer #1: The authors have largely addressed my points through rewording and additional experiments and quantification. 

Minor points:

Lines 206-207: Sox2 on its own does not distinguish quiescent NSCs - this should be phrased differently in the text.

Line 1052 (figure legend): "corpus callosum" not "callous corpus" 

No reference to some of the panels from Figure 5 in the main text?

Reviewer #2: The authors have been responsive to the concerns raised by the reviewers. In particular, they have provided additional data that strongly supports that Gas1(high)+ cells are quiescent neural stem cells in the dorsal lateral SVZ with enhanced gliogenic potential and a significant source of demyelinating oligodendrocytes following cuprizone-mediated demyelination. 

My one remaining concern is the incomplete characterization of fate-mapped cells during remyelination shown in Fig. 7. There is an ~ 5x increase in Olig2+/TdT+ cells from 4 weeks to 10 weeks in the CC. The magnitude of this increase is surprising given that substantial re-myelination has likely already occurred by 4 weeks. It would be useful to know whether the total number of TdT+ cells in the CC at 4 weeks and 10 weeks show a comparable increase i.e., is this an increase in TdT+ cell numbers, upregulation of Olig2, or both? A full breakdown of the percentages of TdT+ cells that are OPCs, oligodendrocytes, astrocytes or cannot be classified at 4 and 10 weeks would provide additional context to this result and whether it is due to ongoing recruitment of stem cells or their ongoing differentiation.

# Author comment: Author response to Decision Letter 1

**DOI:** 10.1371/journal.pbio.3003100.r005

27 Feb 2025

Attachment Submitted filename: Response to Reviewers.docx

# Editor report: Decision Letter 2

**DOI:** 10.1371/journal.pbio.3003100.r006

**Authors:** Lucas Smith

5 Mar 2025

Dear Yuan, 

Thank you for the submission of your revised Research Article "Gas1-high quiescent neural stem cells are multipotent and produce oligodendrocytes during aging and after demyelinating injury" for publication in PLOS Biology and thank you for addressing the last reviewer and editorial requests in this revision. On behalf of my colleagues and the Academic Editor, Mikael Simons, I am pleased to say that we can in principle accept your manuscript for publication, provided you address any remaining formatting and reporting issues. These will be detailed in an email you should receive within 2-3 business days from our colleagues in the journal operations team; no action is required from you until then. Please note that we will not be able to formally accept your manuscript and schedule it for publication until you have completed any requested changes.

**IMPORTANT: As discussed over email, I have updated the 'source data' and 'raw images' files for your manuscripts with the new versions that you provided me. Please do take a moment to double check that everything looks good after these changes. 

Please take a minute to log into Editorial Manager at http://www.editorialmanager.com/pbiology/, click the "Update My Information" link at the top of the page, and update your user information to ensure an efficient production process.

PRESS

We frequently collaborate with press offices. If your institution or institutions have a press office, please notify them about your upcoming paper at this point, to enable them to help maximise its impact. If the press office is planning to promote your findings, we would be grateful if they could coordinate with biologypress@plos.org. If you have previously opted in to the early version process, we ask that you notify us immediately of any press plans so that we may opt out on your behalf.

We also ask that you take this opportunity to read our Embargo Policy regarding the discussion, promotion and media coverage of work that is yet to be published by PLOS. As your manuscript is not yet published, it is bound by the conditions of our Embargo Policy. Please be aware that this policy is in place both to ensure that any press coverage of your article is fully substantiated and to provide a direct link between such coverage and the published work. For full details of our Embargo Policy, please visit http://www.plos.org/about/media-inquiries/embargo-policy/.

Thank you again for choosing PLOS Biology for publication and supporting Open Access publishing. We look forward to publishing your study. 

Sincerely, 

Luke

Lucas Smith, Ph.D.

Senior Editor

PLOS Biology

lsmith@plos.org
